# High-Performance Plasmonic Hafnium Nitride Nanocavity and Nanodisk Arrays for Enhanced Refractometric Sensing

**DOI:** 10.1021/acsami.5c02241

**Published:** 2025-06-05

**Authors:** Beyza Nur Günaydin, Süleyman Çelik, Selim Tanrıseven, Ali Osman Çetinkaya, Fevzi Çakmak Cebeci, Meral Yüce, Hasan Kurt

**Affiliations:** † Faculty of Engineering and Natural Sciences, 52991 Sabanci University, 34956 Istanbul, Turkey; ‡ SUNUM Nanotechnology Research and Application Centre, Sabanci University, 34956 Istanbul, Turkey; § Department of Bioengineering, 4615 Imperial College London, South Kensington Campus, London SW7 2AZ, U.K.

**Keywords:** refractory transition metal nitrides, hafnium nitride, plasmonics, periodic nanoarrays, refractometric sensing, surface lattice resonance

## Abstract

We report on the deposition and thorough characterization of plasmonic hafnium nitride (HfN) thin films, along with the fabrication of HfN nanocavity and nanodisk arrays for refractometric sensing in the visible–near-infrared (Vis–NIR) range. By optimizing reactive RF magnetron sputtering parameters, we achieved high-quality HfN thin films with tunable properties, confirmed through extensive structural, compositional, and optical analyses: grazing-incidence X-ray diffraction, Raman spectroscopy, X-ray photoelectron spectroscopy, Hall-effect measurements, and variable angle spectroscopic ellipsometry. Our optimized HfN films display a gold-like color with metallic behavior down to ∼360 nm, high free-carrier concentration, and minimal energy losses. Using electron-beam lithography, we patterned HfN nanocavity and nanodisk arrays on fused silica substrates. The nanocavity arrays exhibit a grating-coupled surface plasmon polariton (SPP) tunable by adjusting the lattice periodicity, yielding a bulk refractive index sensitivity of up to 636 nm·RIU^–1^ and a figure of merit (FOM) of 17.3. In nanodisk arrays, coupling between LSPR and diffractive orders leads to surface lattice resonances (SLRs), giving rise to narrower spectral line widths and a quality factor exceeding 60. Both array types show significant spectral red-shifts in response to incremental changes in surrounding media refractive indices, demonstrating strong promise for high-performance refractometric sensing. These findings highlight that HfN—a CMOS-compatible, mechanically stable, and cost-effective alternative to noble metals—enables tunable plasmonic devices for biosensing and photonic applications. By bridging a key gap in the exploration of refractory transition metal nitrides, this work emphasizes the potential of HfN in next-generation plasmonic platforms.

## Graphical abstract

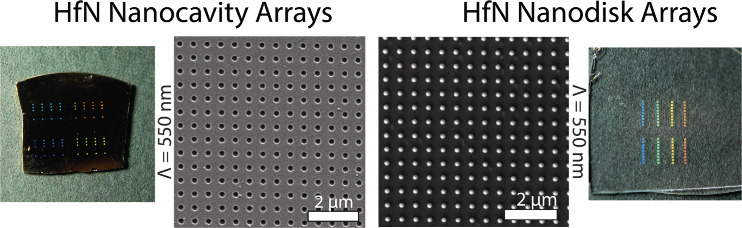

## 1 Introduction

Plasmonic nanostructures can enhance light–matter interactions at the nanoscale by exciting surface plasmons (collective oscillations of free electrons).^1–3^ Unique applications leveraging these plasmon-induced light–matter interactions were highly sought after in the fields of research such as optical biosensors, photocatalysis, solar cells, and super-resolution imaging.^4,5^ It is imperative to engineer the response of a plasmonic nanostructure for a specific application (e.g., matching the resonance wavelength with the absorption profile of a solar cell, optimizing the resonance response of high-sensitivity biosensing, appropriate selection of plasmonic material for application, etc.). The nanostructure geometry and intrinsic dielectric properties of a material play a key role in engineering of these plasmonic structures and their eventual light–matter interaction applications. Although nanostructure geometry provides a wide range of flexibility in engineering such nanostructures, the plasmonic material repertoire is mostly dominated by gold (Au) and silver (Ag).^3,6,7^ They provide strong plasmonic response and chemical resistance yet fail to impress in mechanical robustness and temperature resistance. The refractory transition metal nitrides (TMNs),^3^ such titanium nitride (TiN) and hafnium nitride (HfN), provide a strong alternative, as they have high chemical stability especially for plasmonic applications including aerospace^2^ and microelectronics where they exhibit exceptional mechanical and thermal stability.^3,8,9^ Notably, their key advantage over gold and silver metal is their compatibility with complementary metal oxide semiconductor (CMOS) fabrication processes.^6,7^ Among refractory TMNs, HfN offers tunable optical properties through careful control of deposition parameters governing crystallinity and stoichiometry, unlike noble metals.

Beyond material properties, plasmonic performance is critically influenced by the nanostructure geometry and arrangement. Localized surface plasmon resonances (LSPRs), arising from confined electron oscillations in individual nanoparticles, can be coupled with diffractive orders in periodic arrays to generate collective surface lattice resonances (SLRs). SLRs exhibit ultranarrow spectral line widths, enhanced by dipole–dipole interactions governed by array parameters (e.g., periodicity, particle size), as modeled by coupled dipole approximation (CDA). This interplay between LSPRs and SLRs provides a versatile platform for engineering high-quality resonances in sensing and optical devices.^10,11^

The elevated carrier density, diverse geometries, and precise surrounding medium change sensitivity capabilities of TMNs have resulted in extensive applications in refractometric sensing.^10–15^ We have recently fabricated tunable TiN nanohole arrays that demonstrate improved plasmonic behavior and investigated their extraordinary optical transmission characteristics.^15^ Among various plasmonic transition metal nitrides, HfN has been favored in multiple industries, including coatings, biomedicine, and microelectronics. Recent studies have examined nanostructures with tunable optical properties regulated by thin HfN films and deposition parameters.^16–20^ The optical characteristics of HfN can be modified by adjusting the deposition conditions, which in turn affect the crystal quality and stoichiometry. Nonetheless, the refractory characteristics of TMNs, coupled with the material’s exceptional robustness and chemical inertness, can pose particular manufacturing challenges, particularly when intricate structures such as 3D designs are necessitated.^2^

The geometry and positioning of metal nanoparticles can modify the resonance characteristics of the electromagnetic field in both frequency and dimensions of space. Localized surface plasmons (LSPs) imply the collective oscillation of free electrons in a metallic nanoparticle and the corresponding oscillation of the electromagnetic field.^3,4^ Metal nanoparticles arranged in a periodic arrangement that matches the wavelength of the incoming light can phase-match the scattered fields, reaching a specific particle under certain conditions. The scattered fields are caused by incident light refracted in the array plane. Collective resonances, also known as SLR, can display exceptionally narrow extinction line shapes in uniform arrays of metallic nanoparticles.^21,22^ LSPR in singular nanoparticles and diffractive orders (DOs) present in periodic structures constitute the basis of SLR.^23^ The coupled dipole approximation (CDA) predicts the diffraction of coupled resonances and clarifies their fundamental functions.^21,22,24^ The primary distinction between SLR and LSPR is the inclusion of the dipole sum in the former, as expressed with a set of equations in Supporting Information S15. The dipole sum is dependent upon the arrangement parameters (period, particle size, etc.) and offers an additional degree of freedom to improve the quality of plasmonic SLRs in comparison to LSPRs.^11,21^

In this study, we have optimized HfN thin film deposition using reactive RF-magnetron sputtering and have shown in-depth characterization of the produced HfN thin films for plasmonic applications. We have selected two types of periodic plasmonic structure geometry for nanofabrication: (i) a squarely periodic nanocavity array and (ii) a squarely periodic nanodisk array for assessing plasmonic performance of HfN in the grating coupled surface plasmon and plasmonic surface lattice resonance. We have utilized a hard-mask-based etching-based nanofabrication methodology for nanopatterning HfN thin films. Finally, we assess the plasmon-based refractometric sensitivity performance of HfN using these two plasmon resonance methods.

## 2 Results and Discussion

Our initial experiments focused on investigating the sputtering process of plasmonic HfN thin films. In this context, thin films were fabricated using RF magnetron sputtering under various Ar:N_2_ gas flow ratios indicated in Table S1, and the schematic of the sputtering system is presented in Figure S1. The detailed sputtering parameters for all films are provided in Supporting Information Section S1 and our published article.^15,15^ The sputter-deposited HfN thin films were labeled sequentially from Sample 1 to Sample 7 according to their sputtering conditions (Table S1). The sputtered HfN films exhibited a range of colors across the visible spectrum as the Ar:N_2_ gas flow ratios were varied (Figure 1 and Figure S2). The color variations in the HfN films were investigated in relation to the N-to-Hf ratio and the presence of oxygen as a contaminant. Additionally, visual inspection of the HfN films, Samples 5 and 6, produced with an Ar:N_2_ gas flow ratio of 70:30 (1.2:0.5 sccm Ar:N_2_), exhibits a gold-like color. Figure 1A represents the structural characterization of HfN thin films, including heat maps of grazing incidence X-ray diffraction and Raman spectroscopy measurements. The heat maps present the GIXRD and Raman intensity distribution as a function of 2θ (deg) and Raman shift (cm^–1^) across Samples 1 to 7, respectively. The color scale represents the intensity, with darker shades indicating higher intensities. GIXRD data indicate that all films exhibit polycrystalline structures with a cubic rocksalt phase. Despite their polycrystalline nature, AFM analysis revealed that the surface is exceptionally smooth (1.3 nm RMS, Figure S12). The characteristic HfN peaks were observed at diffraction angles of ∼33.57° (111), ∼39.09° (200), and ∼56.03° (220), consistent with previous HfN studies.^17,25,26^ Notably, the prominent diffraction peak (311) appears only in the plasmonic films, specifically in Sample 5, Sample 6, and Sample 7 (Figure 1A and Figure S3).^16,16^ In the GIXRD results for Sample 3, a HfO_2_ peak was observed at a diffraction angle of 28.5°, which corresponds to monoclinic HfO_2_ (−111).^25^ Additionally, the peak observed at a diffraction angle of approximately 55° corresponds to the forbidden reflection of Si.^27^

**Figure 1:**
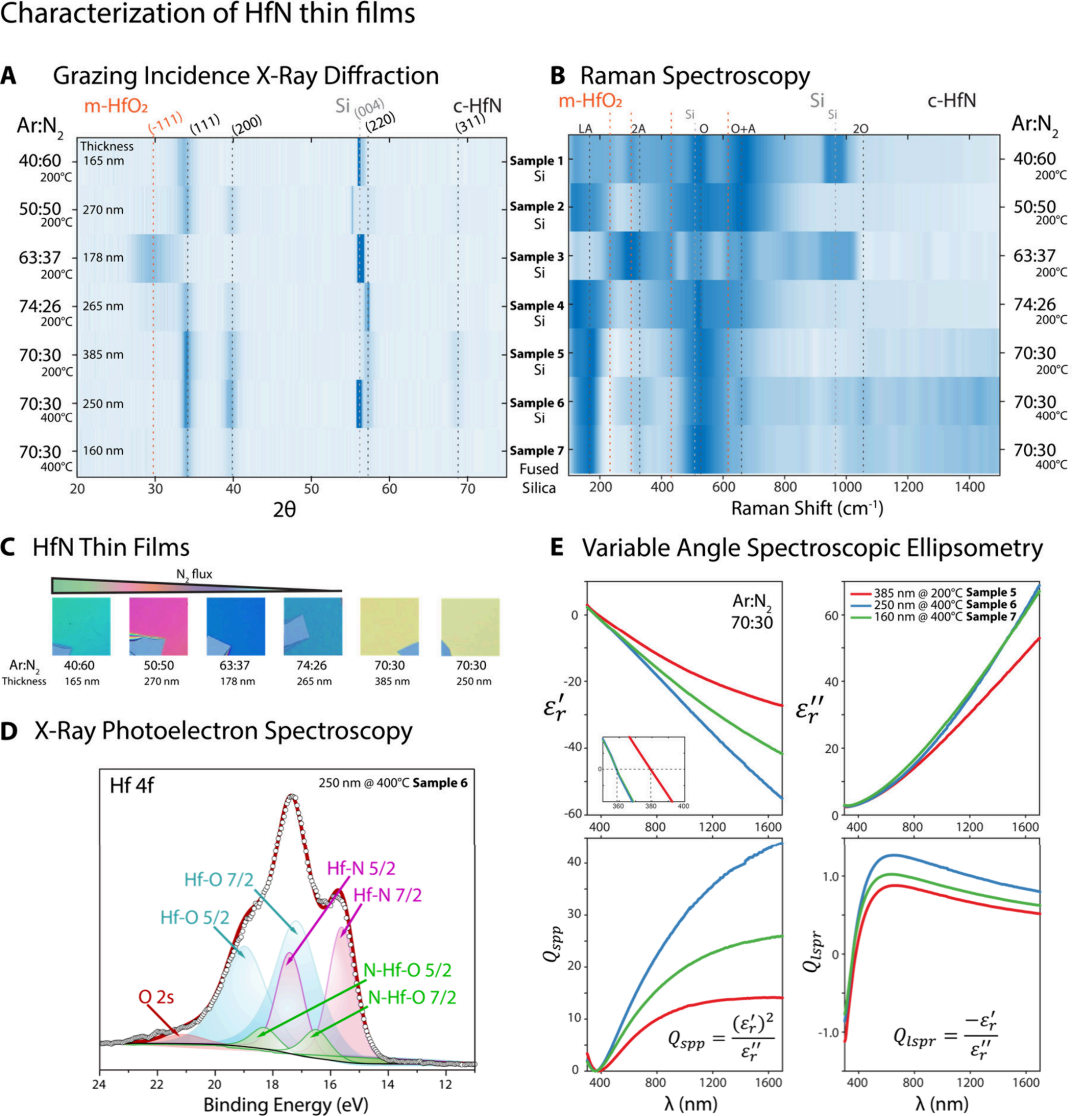
Characterization of HfN thin films, which are deposited under varying Ar:N_2_ flow ratios. (**A**) GIXRD spectra heat map of HfN thin films. The color scale represents the X-ray diffraction intensity. The dashed lines represent the characteristic diffraction planes of monoclinic HfO_2_ (m-HfO_2_) as orange, cubic HfN (c-HfN) as black, and substrate Si wafer as light gray. (**B**) Raman spectra of the HfN thin films. The color scale represents the Raman scattering intensity. The dashed lines represent the characteristic phonon modes of monoclinic HfO_2_ (m-HfO_2_) as orange, cubic HfN (c-HfN) as black, and substrate Si wafer as light gray. (**C**) Photographs of HfN thin films grown on 10 mm × 10 mm Si (100) substrates with varying Ar:N_2_ flow rate ratios. (**D**) The X-ray photoelectron spectroscopy (XPS) of Sample 6, which has a thickness of 250 nm and was grown at a temperature of 400 °C. The high-resolution XPS scan of the Hf 4f region was deconvoluted into components corresponding to Hf–O, Hf–N, and N–Hf–O bonds. The white dots represent binding energy readings. The red curve represents a fitted curve which contains the corresponding energies of Hf 4f 5/2 and Hf 4f 7/2 orbitals for each bond present. (**E**) The variable angle spectroscopic ellipsometry (VASE). The real (*ε′*) and imaginary (*ε″*) parts of the dielectric function, with an inset focusing on the zero-crossing point of *ε′* in the 350–400 nm range. Real and imaginary parts of the dielectric constant (*ε*_*r*_ = *ε′* + *iε″*) from VASE measurements of HfN thin films on Si (100) substrates. The right panels illustrate the quality factors for surface plasmon polaritons (*Q*_SPP_,(*ε′*)^2^/*ε″*) and localized surface plasmon resonances (*Q*_LSPR_, *ε′*/*ε″*).

The characteristic Raman peaks reveal vibrational modes associated with specific bonding and structural features in each sample. Raman peaks at positions 521 cm^–1^ and 935 −990 cm^–1^ were assigned to the Si substrate.^28^ The Si wafer peaks in the measurements suggest that Samples 1, 3, and 4 retain transparency. This observation aligns with the GIXRD measurements, where Si wafer peaks were also observed in these samples. Additionally, Samples 1 and 3 exhibit the tetragonal HfO_2_ phase at 237, 309, 430, 618, and 680 cm^–1^ positions.^29,30^ For Samples 5, 6, and 7, the Raman spectrum confirms the wide phonon bandgap characteristic of HfN thin films, attributed to the significant atomic mass difference (*m*_Hf_:*m*_N_ = 178:14).^16^ The spectrum includes the first-order acoustic band (<200 cm^–1^), a second-order acoustic mode (2A) at 350 cm^–1^, the first-order optical band (530 cm^–1^), and several second-order combinations. These combinations feature an optical and acoustic band shoulder at 660 cm^–1^ and a second-order optical mode (2O) at 1035 cm^–1^, corresponding to HfN bonding in Figure 1B.^16,26^ In Samples 2 and 4, both the first-order acoustic band (<200 cm^–1^) and the first-order optical band (520 cm^–1^) are observed. These films also display a HfO_2_ peak at 618 cm^–1^.^29,30^ The individual Raman analysis graphs for each sample are presented in the Supporting Information, Figure S4.

Considering the analysis results, Sample 6 was identified as the optimal thin film; therefore, the XPS results for Sample 6 are presented in Figure 1D, while the XPS data for all remaining samples are provided in the Supporting Information, Figure S9. A broad survey spectrum covers a wide range of binding energies, revealing the surface composition of the HfN thin films. Distinct peaks for Hf, N, and O are clearly observed. The presence of Hf 4f, Hf 4d, Hf 4p, and Hf 4s peaks reflects the characteristic binding energies of Hf, confirming its incorporation in the sample.^31^ To clarify the contribution of each component in the composition of HfN thin films, we deconvoluted the spectra in the Hf 4f, N 1s, and O 1s regions. Notably, the deconvoluted spectrum in the Hf 4f region comprises three doublets corresponding to the Hf 4f7/2 and Hf 4f5/2 core levels. Peaks labeled Hf–O 5/2 (19.02 eV), Hf–O 7/2 (17.19 eV); Hf–N 5/2 (17.42 eV), Hf–N 7/2 (15.60 eV); N–Hf–O 5/2 (18.28 eV), and N–Hf–O 7/2 (16.51 eV) correspond to Hf bonded with oxygen or nitrogen and oxygen and nitrogen, respectively, with the 5/2 and 7/2 levels resulting from spin–orbit coupling, characteristic of Hf 4f orbitals.^31–36^ The presence of Hf–O peaks suggests hafnium oxides or surface oxidation, while the Hf–N peaks confirm hafnium’s bonding with nitrogen within the HfN compound, providing insight into the oxidation states and the chemical environment surrounding Hf in the sample. In addition, the O 2s peak (21.12 eV) indicates an overlap with Hf states. Further details of O 1s and N 1s spectra are presented as shown in Figure S9. In Sample 4 in Figure S9, the XPS spectra reveal the emergence of distinct Hf–Hf 5/2 (16.9 eV) and 7/2 (15.0 eV) peaks, which can be attributed to the high argon gas flow used during the film deposition process. This elevated argon flow likely influenced the sputtering dynamics, leading to enhanced metallic bonding between hafnium atoms and thereby resulting in the observed peaks.

Optical properties of HfN thin films in the Vis–NIR region were analyzed using a variable angle spectroscopic ellipsometer (VASE) (see Supporting Information Sections 5 and 6 for more details). The Ψ and Δ graphs at 65°, 70°, and 75° (Figure S7) indicate that Samples 1 to 4 exhibit dielectric behavior. However, results demonstrate that HfN thin films display a highly metallic, gold-like color, corresponding to Samples 5, 6, and 7, and possess tunable plasmonic behavior in the Vis–NIR region, as shown in Figure 1E and Figure S7.^37^ The *ε′* = 0 values are called screened plasma frequency (*ω*_*p*_), with a zero-crossing point shown in the inset between 360 and 400 nm, highlighting the transition from dielectric to metallic behavior. While Sample 5 exhibits metallic behavior at ∼379 nm, Samples 6 and 7, with better ellipsometry results, transition to metallic behavior earlier, at around ∼358 nm.

The dielectric constants were fitted using the Drude–Lorentz model, which accounts for contributions from free electrons (intraband transition absorption, represented by the Drude component) and bound electrons (interband transition absorption, represented by the Lorentz component).^38–40^ The Drude–Lorentz model can be expressed as follows:

$$\varepsilon^{\prime }\left(\omega \right)+i\varepsilon^{\prime \prime }(\omega )=\varepsilon_{\infty }-\frac{\omega_{pu}^{2}}{\omega^{2}-i\Gamma_{D}}+\overset{n}{\underset{j=1}{\sum }}\frac{f_{j}\omega_{0j}^{2}}{\omega_{oj}^{2}-\omega^{2}+i\gamma_{j}\omega }$$ 1

2 $$\omega_{pu}=\sqrt{\frac{e^{2}N_{e}}{\epsilon_{0}m^{*}}}\;where\;N=\frac{\sigma_{0}m^{*}}{e^{2}\tau }$$

By using one Drude and two Lorentz oscillators, a suitable fit was achieved for Samples 5, 6, and 7, which exhibit metallic behavior. The fitting results, detailed in Table S2, Supporting Information, show consistent variations in Lorentz and Drude parameters across the samples, demonstrating a match between the experimental ellipsometry spectra and the theoretical model. This consistency underscores the reliability of the fitting process and highlights the structural and electronic transitions influencing each sample’s optical characteristics. Using eqs 1 and 2, along with the values for *e*, σ_0_, and m*, τ, Γ_*D*_, *N*_*e*_ and *ω*_*pu*_ in HfN films were determined.

According to Table S2, *ε*_∞_ denotes the high-frequency dielectric constant, reflecting the contribution of bound electrons and the lattice structure. Higher *ε*_∞_ values, as observed in Sample 6, indicate an enhanced polarization response at high frequencies. The free carrier concentration *N* (cm^–3^) affects the plasmonic response, with higher values, such as those in Sample 6, conferring a stronger metallic character and shifting the plasmon frequency, thus amplifying the plasmonic resonance. Moreover, the plasma energy *E*_*pu*_, which corresponds to the energy of collective electron oscillations, is also highest in Sample 6, suggesting it possesses the most robust plasmonic characteristics. Relaxation time, τ, also indicating the average interval between electron collisions, is longer in Sample 6, signaling reduced losses and sharper plasmonic peaks. Finally, the damping factor Γ, which measures energy dissipation, is lower in Sample 6, minimizing energy loss and further enhancing the resonance sharpness and plasmonic efficiency. In summary, consistent with the quality factor results, Sample 6 exhibits optimal values across all parameters, establishing it as the most promising candidate for applications demanding strong and efficient plasmonic responses.

The ellipsometry results of the HfN thin films investigated in Figure 1E and Figure S7 demonstrate that the dielectric permittivity results were successfully obtained from the 70:30 (Ar:N_2_) flow ratio. The trends in *ε″* reveal how much energy is dissipated within the material at different wavelengths. Ellipsometry results indicate that although Sample 5 exhibits the lowest losses, it does not achieve a high figure of merit due to its lack of a sufficiently high *ε′* value. The ellipsometry results indicate that the optimal sample, which is called Sample 6, has a *ε′* value of −58 at a wavelength of 1700 nm. These plasmonic HfN thin films demonstrate plasmonic performance comparable to previous studies on HfN thin films within the Vis–NIR spectrum, shown in Figure S8.^16,40–42^ *Q*_*Spp*_ and *Q*_*Lspr*_ can be used to assess the quality factor of a plasmonic application for HfN films. The plasmonic figures of merit parameters, both *Q*_*Spp*_ and *Q*_*Lspr*__,_ were indicated. The propagation length of surface plasmon polaritons (SPP) is measured using the *Q*_SPP_ factor, and the field enhancement and refractive index sensitivity of LSPR at the metal/dielectric interface are measured using the *Q*_*Lspr*_ factor. According to *Q*_*Spp*_ and *Q*_*Lspr*_, both values are figures of merit for HfN thin films; Sample 6 shows a higher plasmonic property in the Vis–NIR spectra, which could be advantageous for applications requiring strong and localized electromagnetic field enhancement, such as sensing.

Additionally, the Hall effect measurements of four HfN thin films, in conjunction with the sputtering parameters, provide valuable insights into how the deposition conditions influence their electrical properties. Due to the presence of HfO_2_ peaks indicated in the Raman, GIXRD, and SEM-EDS results, Samples 1 and 3 exhibited high resistivity, preventing accurate measurements. The samples were prepared with varying Ar:N_2_ flow ratios and deposition temperatures, which had a significant impact on carrier concentration, mobility, and resistivity, as shown in Table S3. The observed tunability of the films’ optical properties is attributed to a transition from nitrogen atoms to hafnium atoms, which influences the electronic properties in the following key ways: the density of free electrons, with *V*_*N*_ and *V*_*Hf*_ acting as donor-like and acceptor-like defects, respectively, and the mean free path of free electrons, where *V*_*N*_ and *V*_*Hf*_ serve as primary electron scattering sites in substoichiometric and overstoichiometric films, respectively.^38^

Among the samples, Sample 6, which was deposited under the same conditions as Sample 5 but at a higher substrate temperature of 400 °C, stands out due to its significantly higher mobility and lower resistivity. The increased deposition temperature likely enhanced the film’s crystalline quality, reducing scattering sites and improving charge transport. The higher mobility and moderate carrier concentration suggest that this film benefits from improved nitrogen incorporation and fewer defects due to the elevated temperature. Consistent with the ellipsometry results, Sample 6 exhibits the best electrical properties, making it highly suitable for applications requiring efficient charge transport and high conductivity.

Sample 7 was specifically fabricated for electron beam lithography (EBL) processes, with the corresponding film deposition parameters detailed in Figure S10. The tailored deposition conditions for this sample were optimized to ensure compatibility with EBL requirements, highlighting the precise control over the film’s structural and compositional properties necessary for such advanced lithographic techniques. Figure S11 illustrates the design parameters of the simulations for the HfN nanocavity arrays. Parameters include the periodicity (Λ), hole radius (*r*_*hole*_), and hole depth (*h*_*hole*_). The refractive index of the surrounding medium *n*_*medium*_ is set to 1.0 (air). These structural parameters are crucial for determining the reflectance of the arrays. Figure S11 displays the normalized reflectance spectra of the HfN nanocavity arrays as a function of wavelength (λ) for various lattice periodicities, hole depths, and hole radii. The top graph indicates reflectance as a function of Λ (400–750 nm), while the middle and bottom graphs show reflectance as a function of *r*_*hole*_ and *h*_*hole*_. The reflectance curves reveal how periodicity and depth significantly impact the resonance properties, which are critical for plasmonic applications, as these structural adjustments can tune the plasmonic resonances to the desired wavelengths. As periodicity increases, the resonance wavelength red-shifts, accompanied by a decrease in peak intensity. Examining the hole radius, it is observed that an intense peak is achieved after 90 nm with minimal shifts in the center wavelength occurring beyond a radius of 110 nm. Regarding the hole depth for the nanocavity, it is noted that when the depth is 0 nm (thin film), no SPP reflectance dip is observed. An intense reflectance peak emerges after a depth of 20 nm, and as the depth increases, the fwhm values of the peaks broaden. Based on these simulation results, it was decided to fabricate arrays with a hole radius of 110 nm, a depth of 100 nm, and periodicities of 500, 550, and 600 nm. In light of the *Q*_*Lspr*_ results, Sample 7, with a thickness of 160 nm, was selected as a plasmonic thin film for nanocavity array fabrication on fused silica. The specified simulation parameters were similarly utilized for the nanodisk arrays, and nanodisk arrays with a diameter of 210 nm were conducted over a period of 500, 550, and 600 nm. The schematic diagram illustrates the step-by-step fabrication process of the HfN nanocavity and nanodisk arrays on fused silica using EBL, as shown in Figure 2. This process enables the precise structuring of HfN, which is critical for tuning its optical and plasmonic properties. The details about the design and fabrication of the HfN nanocavity and nanodisk arrays can be found in the Experimental Section and Supporting Information.

**Figure 2:**
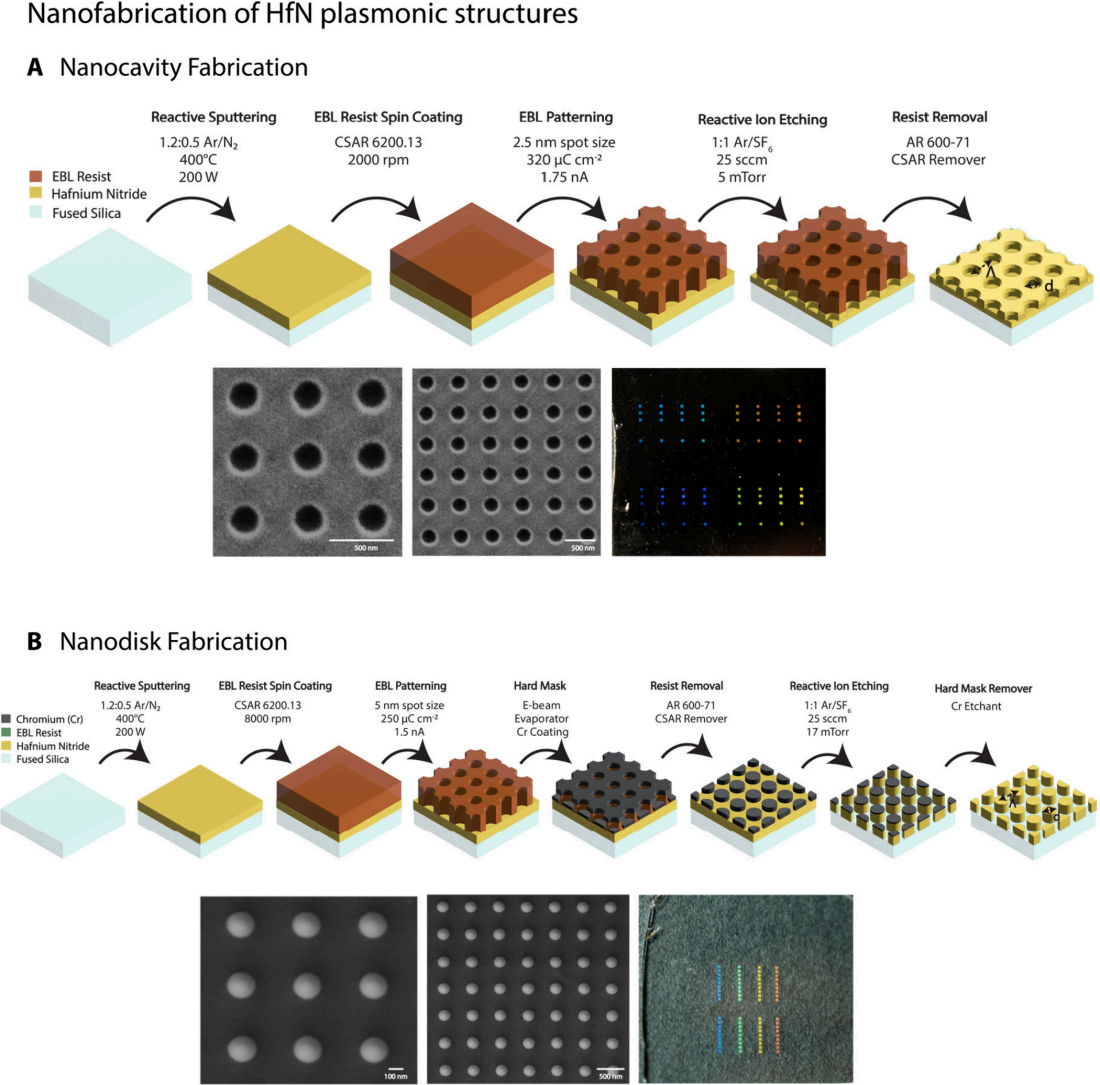
Schematic fabrication processes of (**A**) HfN nanocavity arrays and (**B**) nanodisk arrays, including reactive sputtering, EBL resist spin coating, EBL patterning, Cr hard mask coating (only for nanodisk arrays), reactive ion etching, and resist removal. SEM images showcasing the fabricated HfN nanocavity and nanodisk arrays and photographs highlighting the HfN nanocavity and nanodisk arrays on real substrates. Λ is the periodicity of arrays, and *d* is the diameter of the nanocavity and the nanodisks.

The SEM images in Figure 3 show HfN nanocavity and nanodisk arrays with three different lattice periodicities: 500, 550, and 600 nm. These images reveal the uniformity and structure of the nanocavities and nanodisks, which are essential for consistent plasmonic and optical properties. Each array consists of circular holes with a 110 nm radius for HfN nanocavity arrays and cone disks with a 105 nm radius for HfN nanodisk arrays, whose arrangement affects the resonance properties by altering the interaction between incident light and the surface plasmon modes. The uniform spacing and size of the nanostructures across the arrays demonstrate a successful fabrication with controlled periodicities. The inset images in each SEM provide a wider morphological view of the nanostructure array, illustrating the overall layout and arrangement consistency within the arrays. Furthermore, the angled and sidewall views of HfN nanocavity arrays generated via FIB-SEM are displayed in Figure S12. By evaluation of the morphologies, nanocavities are generated that are uniformly distributed across the surface and have a depth of approximately 100 nm. Despite HfN being classified as a highly durable conductive ceramic, an examination of the sidewall views reveals the formation of nanocavities resembling a nearly fully anisotropic profile. The simulations of the nanocavity and nanodisk array cross-sectional plane (*x*–*z*) in air medium are based on measurements obtained from the SEM images of the fabricated HfN nanostructure arrays. Cross sections reveal concentrated electric fields at the HfN/air and HfN/substrate interfaces for the nanocavity and nanodisk arrays, respectively, indicating the presence of localized plasmonic hot spots. The electric field intensity |*E*|^2^ varies with periodicity and nanostructures, demonstrating how structural adjustments influence light–matter interactions within the nanocavity and nanodisk arrays, which is essential for optimizing the design of plasmonic devices.

**Figure 3:**
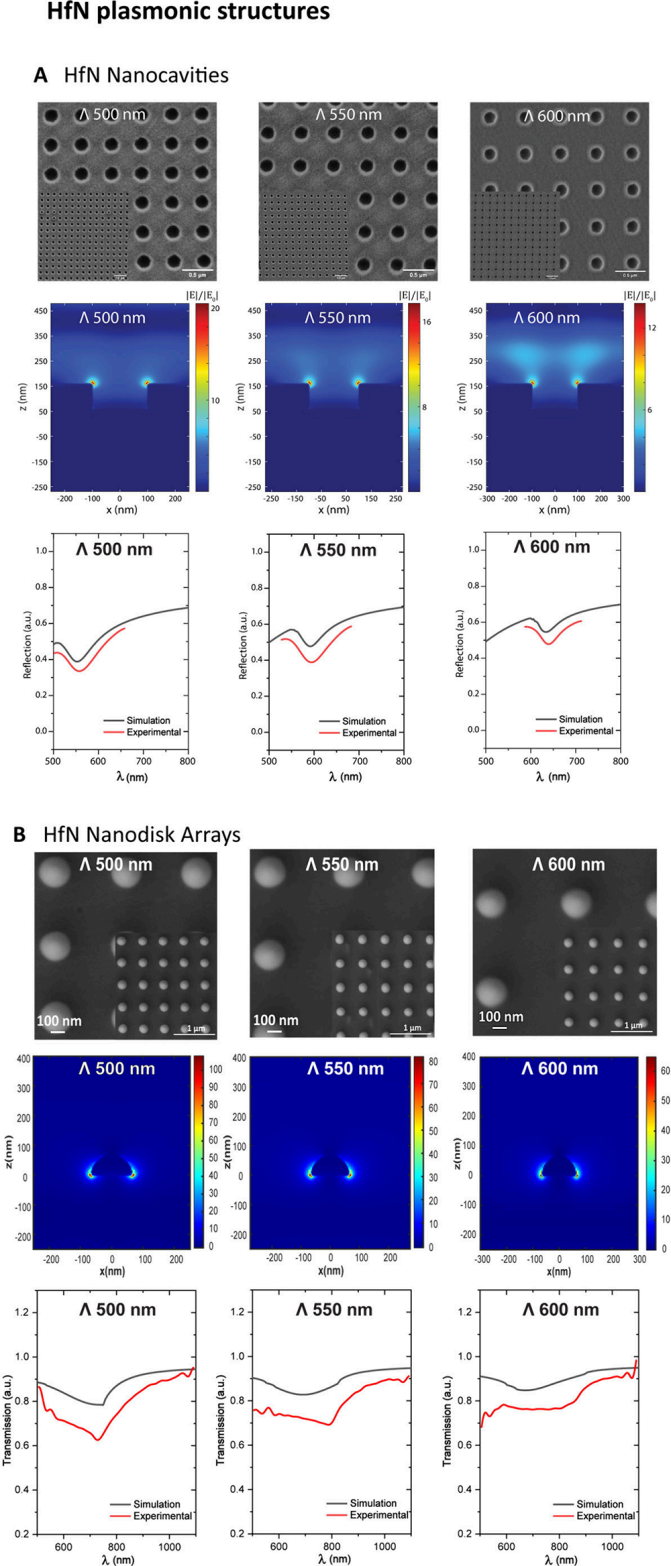
Scanning electron micrographs of nanocavity (**A**) and nanodisk (**B**) arrays were represented concerning periods of 500, 550, and 600 nm, where the scale bar represents 1.0 μm (insets), 0.5 μm (nanocavity arrays), and 100 nm (nanodisk arrays). Distributions of the electric field intensity |*E*|^2^ in the nanocavity and nanodisk arrays’ cross-sectional plane (*x*–*z*) belong to the 500, 550, and 600 nm periods at the HfN/air and HfN/substrate interface, respectively. The reflection and transmission spectra of HfN nanocavity and nanodisk arrays represent a fabricated period in air medium, respectively. The black lines indicate the FDTD simulations of the arrays for the relevant period, whereas the red lines exhibit experimental reflection and transmission spectra.

To accurately excite surface plasmon polaritons and induce the SPP and SLR phenomena, a custom microspectroscopy setup was employed to ensure plane wave illumination from the top and bottom, respectively. The schematic of this custom reflection and transmission microspectroscopy setup is shown in our published manuscript.^15^ The arrays were visualized through the microscope’s imaging arm, which incorporated a monochromatic CMOS camera. Additionally, the spectral resolution of the arrays was achieved by using an imaging spectrometer coupled with an EMCCD camera. The setup includes an imaging spectrometer, various lenses (achromatic doublets), and beamsplitters to focus and direct collimated broad-band light onto the sample. An EMCCD camera captures the reflectance and transmittance signals, which are then analyzed. The setup shows the reflectance and transmittance spectrum, where a shift in the refractive index (*n* to *n* + *Δn*) results in a change in the reflectance and transmittance profiles, as shown in Figure 3. Additionally, a dark band appears in the reflected and transmitted light at the plasmonic resonance wavelength during the measurements. This setup allows for precise spectral measurements and is essential for studying the plasmonic properties of the nanocavity and nanodisk arrays. The simulated and experimental spectra for nanocavity and nanodisk arrays have periodicities of 500, 550, and 600 nm between 500 and 800 nm wavelengths and 500 and 1100 nm in air medium, respectively. Plasmonic resonance was detected in the air medium of the 500 nm period at wavelengths of 575 and 730 nm for nanocavity and nanodisk arrays, respectively. As the period increased, this resulted in a red-shift of the resonance wavelength, which extended to 765 and 856 nm for nanocavity and nanodisk arrays, respectively. The black line represents the simulation, while the red line shows the experimental data. In each graph, the resonance dips in the reflectance and transmittance spectra align closely between the simulation and experiment, confirming the accuracy of the model. The periodicity-dependent shifts in resonance reflect the effect of structural modifications on plasmonic behavior, providing insights into tuning optical responses by adjusting the lattice periodicity. The results show that changes in periodicity directly influence the plasmonic resonances, which can be accurately modeled through simulations, validating the design and potential applications of these arrays in plasmonic devices.

An electromagnetic wave absorbed by the surrounding medium creates an electric field, which can be used as a probe to detect changes in the refractive index of different media. In this study, aqueous solutions of glycerol at varying concentrations (DI water and 1.0, 3.0, 5.0, 10.0, 20.0, 30.0, 40.0, and 50.0 wt % in DI water) were used to demonstrate the detection sensitivity of the LSPR sensor. The Brix refractive index (*n*) results for each prepared medium are provided in Table S4. Due to differences in refractive index, the presence of a dark band can be observed in the reflected and transmitted light at the plasmonic resonance wavelength, as depicted in Figure 4, Figure 5, and Figure 6. Measurements were conducted on 8 distinct arrays for each nanostructure within each medium, and the resonance wavelengths recorded in various glycerol mediums for each nanostructure across all arrays are indicated in Section S13, Supporting Information. The reflectance spectra of HfN nanocavity arrays are shown in Figure 4 with periodicities of 500, 550, and 600 nm, in response to variations in the surrounding medium. The refractive index changes are indicated by different colors, ranging from water to a 50 wt % glycerol–water solution. Each peak in the reflectance spectra represents a plasmonic resonance that shifts as the refractive index of the medium changes, demonstrating the sensitivity of the plasmonic resonance to the surrounding environment. As the glycerol concentration increased, the refractive index of the medium also increased, resulting in a red-shift of the resonance wavelength, which extended from 730 to 765 nm for a period of 500 nm, as seen in Figure 4. Considering the refractive index difference of only 1.5 × 10^–3^, an average 2.87 nm red-shift was observed in the plasmonic resonance wavelength between DI water and 1.0 wt % glycerol for a 500 nm period. The measurements of each array in all media for the bulk-sensitive calculation were analyzed, and the linear regression is presented in Figure 4. Comprehensive linear regression values for each array are included in Supporting Information Section S13, and the highest bulk sensitivity (*S*_*B*_) is determined to be 466 nm·RIU^–1^. The *S*_*B*_, the fundamental sensitivity parameter in refractometric sensing, is calculated by determining the response (*Δλ*) per refractive index variation (*Δn*), which is then expressed in refractive index units (RIUs).^4^ Using linear regression analysis, the change in resonance wavelength per unit refractive index change of the medium is calculated, and the *S*_*B*_ is determined by the slope of the *Δλ* vs *Δn* plot (*Δλ*/*Δn*).^43^ The same trend was observed in other periods (period of 550 and 600 nm) as well, with a red-shift as the concentration of the glycerol increased,^25^ as presented in Figure 4 and for more details Supporting Information Section S13 (Tables S4–S8). Considering the difference in the refractive index of approximately 1.5 × 10^–3^ between 1.0 wt % glycerol and DI water, average ultrasensitive shifting was observed in the arrays with periods of 550 and 600 nm as 1.70 and 1.71 nm, respectively.

**Figure 4:**
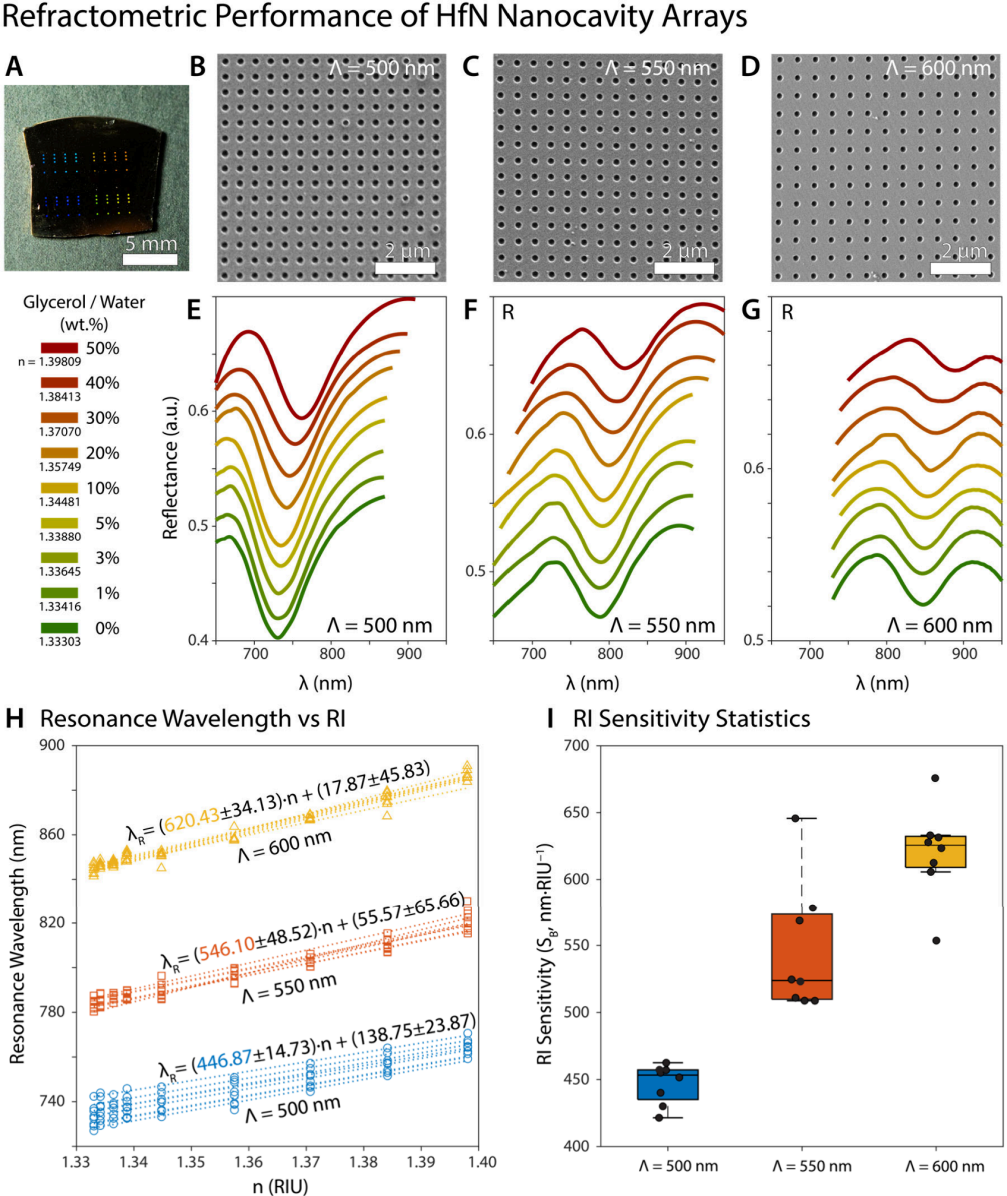
Refractometric performance of HfN nanocavity arrays. (**A**) The photograph of nanofabricated HfN nanocavity arrays with varying periods. (**B**) The SEM micrograph of HfN nanocavity arrays with a period of 500 nm. (**C**) The SEM micrograph of HfN nanocavity arrays with a period of 550 nm. (**D**) The SEM micrograph of HfN nanocavity arrays with a period of 600 nm. The reflectance spectra of HfN nanocavity arrays with periods of (**E**) 500 nm, (**F**) 550 nm, and (**G**) 600 nm in simulated refractive indices of the surrounding medium (varying wt % of glycerol/water mixture). (**H**) The refractive index (RI) sensitivities of each array are investigated. The slope of the linear fit shows the refractive index sensitivity. (**I**) Box plot of the RI sensitivity of the arrays investigated. The black dots represent the RI sensitivity of each individual array investigated.

**Figure 5:**
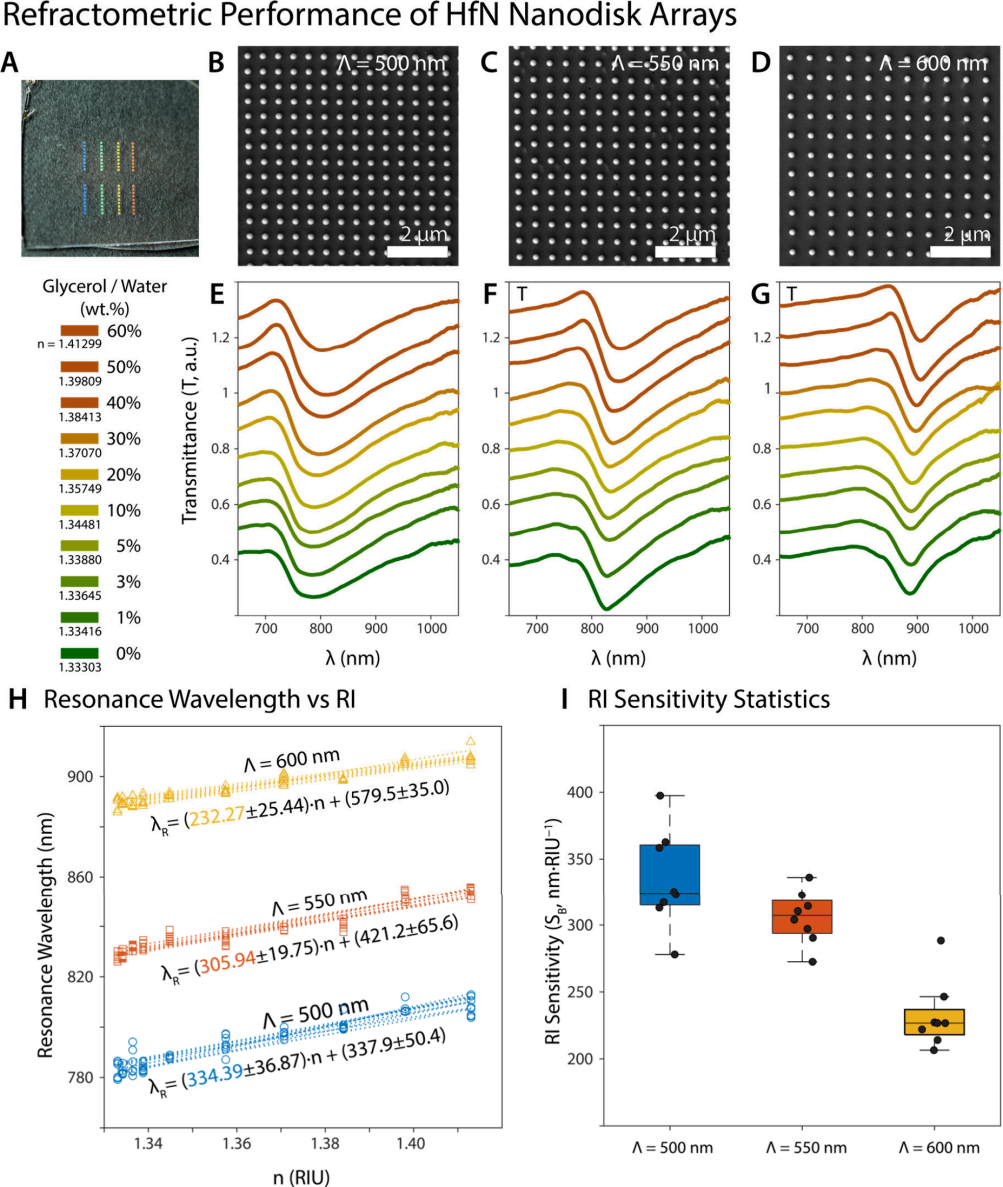
Refractometric performance of HfN nanodisk arrays. (**A**) The photograph of nanofabricated HfN nanodisk arrays with varying periods. (**B**) The SEM micrograph of HfN nanodisk arrays with a period of 500 nm. (**C**) The SEM micrograph of HfN nanodisk arrays with a period of 550 nm. (**D**) The SEM micrograph of HfN nanodisk arrays with a period of 600 nm. The transmittance spectra of a HfN nanodisk array with a period of (**E**) 500 nm, (**F**) 550 nm, and (**G**) 600 nm in simulated refractive indices of the surrounding medium (varying wt % of the glycerol/water mixture). (**H**) The refractive index (RI) sensitivities of each array investigated. The slope of the linear fit shows the refractive index sensitivity. (**I**) Box-chart plot of RI sensitivity of the arrays investigated. The black dots represent the RI sensitivity of each array investigated.

**Figure 6:**
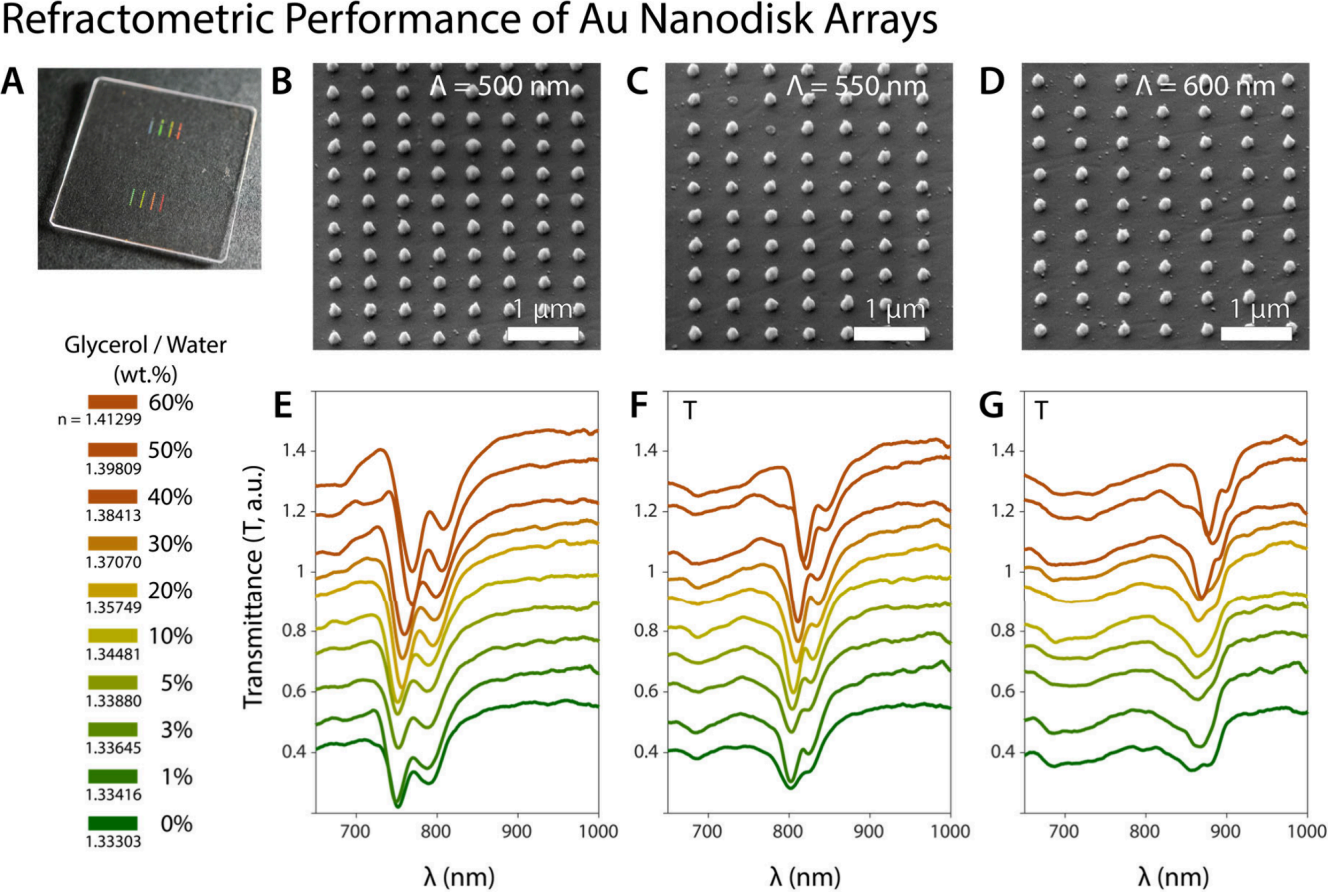
Refractometric performance of Au nanodisk arrays. (**A**) The photograph of nanofabricated Au nanodisk arrays with varying periods. (**B**) The SEM micrograph of Au nanodisk arrays with a period of 500 nm. (**C**) The SEM micrograph of Au nanodisk arrays with a period of 550 nm. (**D**) The SEM micrograph of Au nanodisk arrays with a period of 600 nm. The transmittance spectra of Au nanodisk arrays with periods of (**E**) 500 nm, (**F**) 550, and (**G**) 600 nm in simulated refractive indices of the surrounding medium (varying wt % of glycerol/water mixture).

The reflectance spectra of HfN nanocavity arrays with periods (Λ) of 500, 550, and 600 nm in different *n*_*medium*_ were obsesrved. The refractive index sensitivity of plasmonic HfN nanocavity arrays with periods of 500, 550, and 600 nm for the varying refractive indices was obtained. The spectral measurements were obtained from eight identically constructed 200 μm × 200 μm nanocavity arrays. The sensitivity was calculated from the slope of the linear regression according to the center wavelength vs refractive index.

The enhancement of the refractometric sensitivity value in direct proportion to the center wavelength shifts as the period increases, and the highest sensitivity is successfully achieved at about 636 nm·RIU^–1^ for the HfN plasmonic nanocavity array with a period of 600 nm. In the comparative investigation of refractive indices of various plasmonic materials, TiN nanohole arrays were fabricated, and a low bulk refractive index sensitivity of 180 nm·RIU^–1^ was demonstrated in our previous publication due to the low plasmonic quality factor of TiN thin films.^15^ Furthermore, Shkondin et al. developed TiN trench (grating) structures, achieving a sensitivity of 430 nm·RIU^–1^ because of the high aspect ratio grating sensor that possesses Rayleigh–Wood anomalies.^44^ In contrast, Cetin et al. demonstrated a sensitivity of 671 nm·RIU^–1^ for Au NHAs because of utilizing a hybrid substrate, including a high “*n*” dielectric interlayer over a transparent substrate.^45^ Shokova et al. carried out a simulation study on nanohole arrays with different parameters to demonstrate the effect of a Au–silica–Au optical cavity on sensing performance and achieved a bulk sensitivity of 275 nm·RIU^–1^.^46^ Kurt et al. developed PANTOMIM Au NHAs with a bulk sensitivity of approximately 570 nm·RIU^–1^ in a metal–insulator–metal (MIM) structure. The main reason these PANTOMIM chips achieve high sensitivity is that the system incorporates the advantages of plasmonic nanohole arrays—such as tunable periodicity, coupled plasmonic response, and extraordinary optical transmission—while also reducing the limitations associated with thin film plasmonic metals. The detection of *Escherichia coli* bacteria has been carried out with high precision, especially in urine samples, by PANTOMIM.^47^ Dormeny et al. also presented a study focused on the design and simulation of a refractive index sensor employing gold nanostructures. The sensitivity of optimum design (20 nm nanowire supported thin film) was achieved as 1916 nm·RIU^–1^ because of the coexcitation of SPR and LSPR modes.^48^ Overall, it can be said that gold nanohole array structures possess bulk refractive index sensing between about 200 nm·RIU^–1^ and 2000 nm·RIU^–1^.^45–49^ The bulk refractive index of our HfN nanocavity arrays is within the gold range, specifically at 636 nm·RIU^–1^, thus allowing for comparison with Au and considering it an alternative material. This behavior is critical for sensing applications, where shifts in resonance can be used to detect changes in the refractive index of nearby substances. Sensitivity increases with periodicity, suggesting that larger periodicities provide greater shifts in resonance wavelength in response to surrounding changes, which is valuable for high-sensitivity sensing applications.

Following the sensitivity calculation, the figure of merit (FOM)^43^ was determined using eq 3:^45^

3 $$FOM=\frac{S_{B}}{FWHM}$$

The FOM is established by analyzing the relationship between the full width at half-maximum (fwhm) and *S*_*B*_. When comparing the effectiveness of various biosensor systems, FOM is the most frequently used metric. However, it is important to note that the resonant wavelength, grating structure, and metal film all play a critical role in determining the FOM value.^43^ The fwhm represents the width of the plasmonic resonance peak in the spectral response of a plasmonic sensor; a narrower fwhm generally indicates higher sensitivity, as it corresponds to a sharper and more distinct resonance peak, facilitating more precise detection of refractive index changes. fwhm values decrease as the plasmonic resonance wavelength increases, with the lowest fwhm recorded as 36.8 nm for a periodicity of 600 nm. Based on the lowest fwhm value for each periodicity, the highest FOM achieved is approximately 17.3 for HfN nanocavity arrays with a period of 600 nm. The relatively wide fwhm of our plasmonic array is primarily responsible for the low FOM value observed in this study. The inherent optical losses of HfN primarily contribute to the observed broad resonance behavior in HfN nanodisk arrays. In comparison to gold, HfN exhibits a larger imaginary component of its dielectric constant, resulting in increased damping of plasmonic modes and, subsequently, broader resonance peaks. Depositing plasmonic HfN thin films with diminished loss and enhanced quality factors can yield sharper peaks. Furthermore, HfN nanodisk arrays were fabricated to reduce the broad fwhm values observed in the nanocavity, resulting in a notable peak narrowing. Consequently, the fwhm value can be diminished by modifying various designs, periods, and nanopattern dimensions. Ultimately, any contamination and residual resist on the nanostructures generated during the nanofabrication process diminish plasmonic performance, resulting in peak broadening.

HfN nanodisk arrays were fabricated to decrease the fwhm value, and the SLR characteristics of nanodisk arrays with varying lattice spacings were investigated. Due to the elevated fwhm values in LSPR graphs, a viable approach to improve the quality of LPRs is to utilize radiative far-field coupling in regular nanoparticle arrays through their scattered radiation fields.^22^ In comparison to SLR, LSPR demonstrates a greater fwhm, thereby constraining its application in devices reliant on high quality factors. If a diffracted wave propagates in a plane, it may interact with individual nanoparticles of the LSPR, resulting in the observation of SLR characteristics. Figure 5 illustrates that as the refractive index of the medium increases from 1.33 to 1.4 RIU, the SLR peak for each period exhibits a red-shift. HfN nanodisk arrays with a 500 nm periodicity demonstrate a plasmonic resonance wavelength of 778 nm in water; however, as the refractive index increases (50 wt % glycerol), the resonance red-shifts to 812 nm. At the 600 nm period, the nanodisk arrays indicated resonance at 878 nm in water, and with an increase in glycerol concentration, they displayed resonance in the NIR region at 905 nm with 50 wt % glycerol (Tables S9–S11). The plasmonic response of the HfN nanocavity arrays demonstrates a linear regression throughout the entire refractive index range (Figure 4). In addition, a linear regression phenomenon is observed when the plasmonic response of the nanodisk arrays is examined as the refractive index. As the SLR characteristic becomes more pronounced at longer wavelengths, the SLR-enhanced linear response strengthens due to mode hybridization and near-field effects. Figure 5 illustrates that as the period increases, the fwhm width decreases, and the dip width reduces from 115 nm at a period of 500 nm to 15 nm at a period of 600 nm.

The quality factor of resonance, *Q*, which relates to the ratio of energy stored to energy dissipated by an oscillator, is calculated as *Q* = λ/fwhm (where λ is the resonance wavelength and fwhm is the resonance width) and increases with the increasing distance between the nanodisk arrays.^50^ The *Q*_LSPR_ value obtained from ellipsometer measurements was approximately 1.3 for HfN. Nevertheless, subsequent to the SLR coupling effect, the quality factor exceeded 60 for HfN nanodisk arrays. The quality factor *Q* is exclusively dictated by the dielectric function of the metal at the plasmon frequency, with *Q*-factors around 10–20 frequently observed for the majority of LSPR.^21,51^

For an assessment of the refractometric performance of HfN nanocavities and nanodisk arrays, Au nanodisk arrays shown in Figure 6 were fabricated. The transmission measurements of Au nanodisk arrays produced at 500, 550, and 600 nm periods, which are similar to HfN, were conducted in environments with varying refractive indices. The SLR dip for the Au nanodisk arrays occurs at a resonance wavelength of 771.11 nm for a period of 500 nm, exhibiting an fwhm of 18 nm, excluding the broad base in the width definition of each dip. As the separation between the nanodisk lattice constant increases, resulting in a longer period, the fwhm values of the dips narrow. For a 600 nm period, the fwhm value significantly decreases to 12.89 nm, and the plasmon line shape exhibits a red-shift to an 880 nm resonance wavelength. Examination of both the HfN and Au nanodisk graphs reveals that the SLR behavior becomes increasingly prominent with an increase in lattice spacing, and the width of the plasmon resonance diminishes.^52^

## 3 Conclusion

This study demonstrates the successful deposition and detailed characterization of plasmonic HfN thin films and nanostructures optimized for Vis–NIR plasmonic applications, particularly in refractometric sensing. Using reactive RF magnetron sputtering with controlled Ar:N_2_ gas flow ratios and substrate temperatures, HfN thin films were deposited on silicon and fused silica substrates and underwent a comprehensive structural analysis. Sample 6, produced with a 70% Ar and 30% N_2_ ratio at an elevated substrate temperature of 400 °C, emerged as the optimal candidate, showing exceptional plasmonic and electronic properties. This sample displayed a cubic rocksalt structure confirmed by GIXRD analysis and minimal surface roughness (1.3 nm RMS). The XPS analysis further identified strong hafnium–nitrogen bonds with minimal surface oxidation, indicating a stable HfN composition. The sample exhibited high free-carrier concentration (2.25 × 10^22^ cm^–3^) and low resistivity (3.60 × 10^–3^ Ω·cm), key parameters for efficient plasmonic performance. Its real dielectric permittivity (*ε′* ≈ −58 at 1700 nm) confirmed a strong plasmonic response, with the Drude–Lorentz fitting showing superior parameters such as a longer relaxation time (τ) and low damping factor (Γ), contributing to minimized energy dissipation. Additionally, HfN nanocavities and nanodisk arrays fabricated on this sample demonstrated LSPR and SLR tunability. Using a custom reflection and transmission microspectroscopy setup, these arrays displayed ultrasensitive red-shifting in the resonance wavelength in response to changes in the surrounding medium, with shifts reaching up to 3.21 nm for a refractive index change of only 1.5 × 10^–3^. Then, these arrays exhibited refractive index sensitivity across different media, achieving a bulk sensitivity of 636 nm·RIU^–1^ and a FOM of 17.3 metrics, highly competitive with traditional plasmonic materials like gold. Upon analyzing the SLR behavior derived from the diffraction coupling of the nanodisk arrays with LSPR, significant enhancements in the optical performance of the HfN nanodisk arrays have been noted. The minimum fwhm value for LSPR was reported at 36.8 nm with a periodicity of 600 nm, whereas in HfN nanodisk arrays, this value diminished to 15 nm. This situation demonstrates that the optical resonances of the HfN nanodisk arrays exhibit significantly sharper characteristics and enhanced quality. The calculations utilizing dielectric constants derived from ellipsometer results indicated that the *Q*_LSPR_ factor, representing the quality of LSPR, was established at 1.3 for the HfN nanodisk arrays. Nevertheless, with the appearance of the SLR coupling effect, this quality factor of LSPR has exceeded 60. This substantial increase signifies that the SLR coupling of HfN nanodisk arrays markedly improves resonance sharpness and quality. Such a high sensitivity level, coupled with the stability and tunability of HfN, makes it a compelling alternative to noble metals for plasmonic applications. Through controlled sputtering conditions and in-depth characterizations, this work establishes a framework for developing HfN-based plasmonic devices, addressing a significant gap in the literature on refractory metal nitrides for advanced photonic applications.

## Experimental Methods

### Thin Film Deposition

The hafnium nitride thin films were deposited on Si (100) and fused silica JGS2 substrates (Microchemicals GmbH, Germany) from a 2 in. diameter 99.9% pure hafnium target (HF-ST-2-6-3N, Testbourne Ltd., UK) using reactive radio frequency (RF) magnetron sputtering (NVSP-400, Nanovak, Turkey) in an ISO 6 class 1,000 cleanroom. Initially, the sputtering chamber was cleaned of oxygen before HfN deposition using DC sputtering of a 2 in. diameter, 99.95% pure Zr target (ZR-ST-2-6-3N5, Testbourne Ltd., UK) at a temperature of 400 °C and an Ar flow rate of 18 sccm. The chamber was pumped down to a base pressure of ∼8 × 10^–6^ Torr before each deposition stage. The reactive RF magnetron sputtering of hafnium nitride thin films took place under varying Ar:N_2_ flow rate ratios with an RF power of 200 W, as illustrated in Figure S1. Initially, the substrate temperature was set to 200 °C in the deposition of initial flow rate optimization studies (samples 1–5). Later, the substrate temperature was set to 400 °C for HfN thin film depositions under a 1.2:0.5 Ar:N_2_ flow rate ratio for Si and fused silica substrates (samples 6 and 7). The detailed sputtering conditions for all films are provided in Supporting Information Section S1.

### Design and Simulation

All parameters affecting the biosensor’s performance were simulated using commercial finite-difference time-domain (FDTD) software. A broadband light source with *x*-axis polarization, spanning 400 to 1100 nm, was selected as the excitation light for the simulation setup. The electric field intensity distributions were recorded on the *xz*-plane cross-section of the nanocavity array, 1 nm above the HfN/air interface, by using a 5 nm uniform mesh. Mesh refinement was achieved by applying the conformal variant 1 method, and an additional custom mesh with 1 nm resolution was added at each interface to ensure accuracy in the metal/dielectric layers. Boundary conditions were set to optimize simulation efficiency, with a steep-angle perfectly matched layer (PML) in the *z*-axis and symmetric and antisymmetric conditions applied along the *y*-axis and *x*-axis, respectively. The auto shut-off level was set to 10^–7^, and the simulation time was fixed at 500 fs. As a result of these simulations, the nanocavity radius was chosen to be 110 nm with a nanocavity array depth of 100 nm, and the arrays were simulated in an air medium with periodicities ranging from 400 to 750 nm.

### Fabrication

The nanopatterning of the thin film was accomplished using EBL (Vistec EBPG 5000+). Figure 2 provides illustrations of the fabrication processes, which cover the sequential steps involved in manufacturing HfN thin films, resist coating, patterning using EBL, and etching. Sample 7, produced on fused silica, was selected for fabricating a periodic nanocavity and nanodisk array with various periodicities. Each period cluster has 16 nanocavity and nanodisk arrays of 200 μm × 200 μm. A baked resist layer with a thickness of 550 nm was produced by first coating a HfN thin film with a CSAR 6200.13 coating at 500 rpm for 5 s and 2000 rpm for 45 s. This coating was then soft-baked for 2 min at 150 °C. Next, using EBL and an electron beam with a 2.5 nm (nanocavity) and 5.0 nm (nanodisk) spot size, 320 μC·cm^–2^ (nanocavity) and 250 μC·cm^–2^ (nanodisk) dose, and 1.75 nA (nanocavity) and 1.5 nA (nanodisk) current, the resist layer was nanopatterned. The remaining CSAR layer structures were developed by applying successive treatments of a developer, 2-propanol:1-methyl isobutyl ketone (1:1), and, finally, nitrogen dried and hard baked for 1 min at 130 °C. Before reactive etching, the Cr hard mask was coated by an e-beam evaporator only on the nanodisk arrays. ICP-RIE (Oxford Plasma Lab System 100) was used to etch the HfN layer of the developed sample for 30 s at 2000 W ICP power, 300 W RF power, 25 sccm of SF_6_ flow, 25 sccm of Ar flow, a 5 mTorr process pressure, and a 10 °C substrate temperature. The residue CSAR resist layer was removed from the HfN layer by dipping the patterned HfN layer in CSAR remover solvent (AR 600-71) for 3 min static and 3 min with sonication to remove any remaining resist, followed by nitrogen stream drying. Optimized parameters were used in our previous publication for the fabrication of Au nanodisk arrays, and unlike HfN nanostructures, nanodisk arrays were obtained by the lift-off method.^11^

### Characterization

With the assistance of a KLA Tencor surface profiler, the thickness of the deposited films was measured. The morphology of the nanopatterns was determined by SEM (Zeiss, Leo Supra VP 35) and FIB-SEM (JEOL JIB-4601 MultiBeam FIB-SEM). Hafnium, nitrogen, and oxygen were examined when conducting SEM-EDX (Zeiss, Leo Supra VP 35) mapping analysis on the deposited material compositions. XRD measurements (Bruker, D8 Advance) were carried out in grazing-incidence GIXRD modes with increments of 0.002° over an angle range of 20 to 80 degrees with a 4 s step time. These measurements provided additional compositional and crystallographic information about thin films. In addition, Raman measurements were taken using a Renishaw InVia Reflex Raman spectrometer with a 532 nm incident laser beam in the 100–1500 cm^–1^ Raman shift range, with a laser power of 10 mW and a 20 s exposure time to determine the phases of the deposited HfN. Using the tapping mode, an AFM (Nanomagnetic Instruments/hpAFM) acquired the surface topography and roughness of the thin film and pattern height of the nanopattern. The scanning electron microscope (Zeiss, Leo Supra VP 35) was able to visualize the detailed surface morphology of the nanocavity arrays. The Hall effect measurements were performed using the NanoMagnetic Instruments LTes-HEMS system, conducting three repetitions at room temperature with the ezHEMS measurement setup to measure the electric properties. XPS measurements were performed by using a Specs Flex mode XPS system with a Phoibos analyzer and a medium area lens (1.5 kV). The experiment was conducted in fixed analyzer transmission mode with a pass energy of 100 eV and an excitation energy of 1486.71 eV. Data acquisition was carried out at room temperature, with a dwell time of 0.2 s per point. The effective work function was set to 4.332 eV, and the detector voltage was 1580 V. Binding energy spectra were collected over a range up to 1387 eV, providing insights into the sample’s chemical composition and electronic states. To analyze the Hf 4f, N 1s, and O 1s regions, the scan was performed in the fixed analyzer transmission mode with a pass energy of 45 eV and an excitation energy of 1486.71 eV. The dwell time was 0.4 s per point, and data were collected in the binding energy range up to 406 eV, capturing 181 data points in a single scan. The X-ray source used was an XR50 M (UXC1000). The data were processed and deconvoluted by using the Casa XPS software, ensuring precise peak fitting and analysis. The Shirley background model was also applied to subtract the baseline signal accurately.

### Drude–Lorentz Fitting of Deposited HfN Thin Films

A variable angle spectroscopic ellipsometer (J.A. Woollam, VASE) system was used to acquire Ψ and Δ data at incident angles of 65°, 70°, and 75° to characterize the optical properties of the deposited thin films. The data from spectroscopic ellipsometry were modeled via the WVASE32 software. The models developed by two Lorentz and a Drude oscillator were applied to the wavelength range of 300–1700 nm to extract the real (*ε′*) and imaginary (*ε″*) dielectric coefficients. The HfN Drude model (from ref 53: pp 368–369; 138–2500 nm), which is in the WVASE32 software, was used to fit the plasmonic HfN thin films exhibited in Figure 1 and Figure S7.^53^

### Spectral Measurement

The reflection and transmission spectra of the HfN plasmonic nanocavity and nanodisk arrays were measured using a custom microspectroscopy setup. As the light source, we utilized a fiber-coupled tungsten halogen lamp (Ocean Optics, HL-2000-HP) and collimated it into the illumination arm of the microscope. After that, the collimated illumination was focused on the back focal plane of an achromat objective (Olympus, PLN 4× with 5.5 mm field of view), employing an achromatic doublet (Thorlabs, AC254-100-AB) housed in a cage system. The light beam spot area on the chip is calculated as 23.8 mm^2^. In our chips, the entire surface is not in the form of an active area with nanopatterns; instead, nanocavity and nanodisk arrays with an active area of 200 μm × 200 μm per period have been produced, as shown in Figure 2. The number of nanopatterns produced within each array varies according to the period.

A motorized XYZ stage manufactured by Sutter Instruments (model number MP-285) was used for the spatial manipulation of the sample. Two broadband, nonpolarizing 50:50 beam splitters (Thorlabs, BS-013) were utilized to physically separate the spectroscopy and imaging components of the microscope. Using an achromatic doublet (Thorlabs AC254-200-AB), the imaging arm was focused on a monochromatic CMOS camera (Basler, model acA4112-30um). Again, utilizing an achromatic doublet (Thorlabs AC254-200-AB), the spectroscopy arm was focused on a 10 μm slit on an astigmatism-free Schmidt–Czerny–Turner imaging spectrometer (Princeton Instruments, IsoPlane SCT 320) with a focal length of 320 mm and grating of 150 lines·mm^–1^.

To conduct the spectral imaging experiments, a back-illuminated EMCCD scientific camera (made by Princeton Instruments and referred to as the ProEM HS:1024BX3) was coupled with the imaging spectrometer. The microspectroscopy system’s wavelength and intensity were calibrated using Hg and Ar/Ne lamps (Princeton Instruments IntelliCal) and a NIST traceable LED-based intensity calibration light source (Princeton Instruments IntelliCal).

## Supplementary Material



## Data Availability

All data are available in the main text or the Supporting Information.

## Notes

S1. Optimization of HfN thin film production at different Ar:N_2_ ratios; S2. XRD analysis; S3. Raman analysis; S4. Stoichiometric analysis of HfN thin films at varying Ar:N_2_ ratios; S5. Optical properties of HfN thin films at varying Ar:N_2_ gas flow ratios; S6. Ellipsometry fitting; S7. Hall measurement; S8. Literature comparison; S9. XPS analysis; S10. Sample 7 (EBL sample) data explanation for HfN thin film on Si wafer and FS substrate; S11. FDTD simulations for HfN nanocavity array; S12. The surface topology of bare HfN film and patterned plasmonic HfN nanocavity array by AFM analysis; S13. Refractometric sensing results of HfN nanostructures at various glycerol concentrations; S14. Au nanodisk array refractometric performance; S15. The coupled dipole approximation (PDF)
