# Supplementary material for: High-Performance Plasmonic Hafnium Nitride Nanocavity and Nanodisk Arrays for Enhanced Refractometric Sensing
Source: ACS Appl Mater Interfaces. 2025 Jun 5;17(24):35842–56. doi: 10.1021/acsami.5c02241 (PMC12186218; doi:10.1021/acsami.5c02241)
Supplement: Supplementary file 1 [file am5c02241_si_001.pdf]

Supporting Information

High-Performance Plasmonic Hafnium Nitride Nanocavity and Nanodisk Arrays for Enhanced Refractometric Sensing

Beyza Nur Günaydin[1,2], Süleyman Çelik[2], Selim Tanrıseven[2], Ali Osman Çetinkaya[2], Fevzi Çakmak Cebeci[1,2], Meral Yüce[2,3]*, Hasan Kurt[3]*

[1] Faculty of Engineering and Natural Sciences, Sabanci University, 34956, Istanbul, Türkiye

[2] SUNUM Nanotechnology Research and Application Centre, Sabanci University, Istanbul, 34956, Türkiye

[3] Department of Bioengineering, Imperial College London, South Kensington Campus, London, SW7 2AZ, UK

***Corresponding authors**: meralyuce@sabanciuniv.edu & h.kurt@imperial.ac.uk

Supporting Information

S1. Optimization of HfN thin film production at different Ar: N_2 ratios.

HfN thin films were deposited under controlled conditions utilizing reactive magnetron sputtering. The RF magnetron sputtering system (Nanovak, NVSP-400) was employed with a 99.9% pure hafnium target (Kurt J. Lesker, USA) in a Class 1000 cleanroom environment. Base pressure was gained using a mechanical pump (up to 10^{-3}) and turbo pump (up to 10^{-6}), and the sputtering chamber was maintained at a base pressure of approximately 8×10^{-6} Torr. Thin films were deposited on silicon (100) wafers and fused silica substrate (only for EBL sample). Prior to thin film deposition, the substrates were ultrasonically cleaned sequentially in acetone, methanol, and ethanol.

Before initiating HfN deposition, reactive DC magnetron sputtering with a zirconium (Zr) target (Kurt J. Lesker, USA) was employed to mitigate residual oxygen levels within the chamber. Detailed procedures regarding the cleaning process with Zr target are available in our previously published article [1]. The oxygen scavenging process utilizing Zr was performed through DC magnetron sputtering, with substrate heating maintained at 400°C under a pure Ar gas flow of 18 sccm and operating pressure of 10^{-4} Torr (Figure S1, Step I). After the DC magnetron process was completed, RF magnetron sputtering for HfN deposition commenced without opening the sputter chamber. Both the DC and RF magnetrons are equipped with shutters; the shutter of the RF magnetron is closed when DC sputtering is conducted, and the shutter of the DC magnetron is closed during RF sputtering, as shown in Figure S1.

The RF magnetron sputtering process was initiated by sputtering a high-purity hafnium target in an Ar atmosphere. To remove unintentional impurities—such as organic contaminants and native oxide layers—present on the surface of the Hf target, a short ion etching procedure (~10 minutes) was conducted using Ar ions prior to deposition. Following this surface cleaning step, a controlled amount of N_2 gas was introduced into the sputtering chamber. Throughout the deposition process, Ar and N_2 gases were delivered into the vacuum chamber via a PID-controlled throttle valve system to ensure precise regulation of the chamber pressure. Stable gas pressure is typically attained within five minutes, after which the shutter above the substrate is opened as shown in Figure S1, Step II. This enabled the deposition of HfN thin films in a stable atmosphere of argon and nitrogen gas flow. The integration of magnetrons and the installation of a shutter system above the substrate markedly diminish the formation of chemical composition gradients in the deposited film, thereby enhancing uniformity. Metal-rich nitrides demonstrate HfN and its conductivity, whereas nitrogen-rich nitrides typically exhibit dielectric characteristics. The magnetron power was maintained at a constant 200 W, and the HfN thin film deposition rate varied between 3 to 5 nm/min at 200°C and 400°C (Figure S1 (Step II) and Table S1). The substrate temperature was monitored using a system of thermocouples positioned within the vacuum chamber and controlled via a control board.

Post-deposition, the presence of HfN films on the substrates was confirmed by a visible change in the color, which varied according to different Ar:N_2 ratios. The process parameters included deposition rates controlled by magnetron power, partial pressures of Ar and N_2, substrate temperatures, and film thickness, as summarized in Table S1.

The fabricated films are labeled sequentially from Sample 1 to Sample 7, and the film thicknesses range between 165 nm and 385 nm.

Sample 6 exhibits the most favorable plasmonic properties, and to achieve a thinner film, Sample 7 was produced under the same sputtering conditions as Sample 6 on silicon (100) wafers and fused silica substrate but with a reduced sputtering duration. All films from Sample 1 to Sample 6 were deposited exclusively on Si wafers, whereas Sample 7 was grown on both fused silica and Si wafers. The HfN thin film grown on fused silica was utilized for electron beam lithography (EBL) to fabricate a plasmonic nanostructures.

Supporting Information

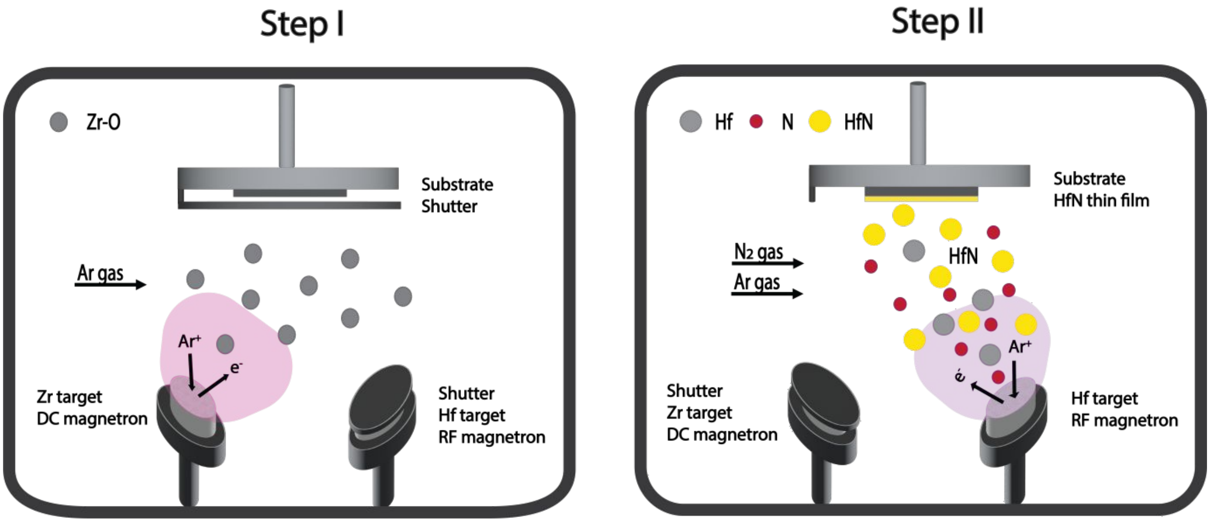

Figure S1. Schematic illustrations of HfN thin film deposition using reactive magnetron sputtering. Step I DC magnetron sputtering of Zr for oxygen scavenging; Step II RF magnetron sputtering of HfN by introducing various Ar:N_2 gas flows.

Table S1. Sputtering parameters and deposition conditions for HfN thin films prepared under varying Ar: N_2 gas flow ratios. The table lists the sample numbers, Ar: N_2 gas flow rates (sccm), gas flow ratios (%), base pressure (Torr), substrate temperature (°C), deposition pressure (Torr), RF power (W), and resulting film thicknesses (nm).

Sample No	Ar: N_2 (sccm)	Ar: N_2 (%)	Base pressure (Torr)	Substrate Temp. (°C)	Deposition Pressure (Torr)	RF power (W)	Thickness (nm)	Substrate
1	1.0: 1.5	40: 60	10^{-6}	200	10^{-3}	200	165	Si wafer
2	1.0: 1.0	50: 50	10^{-6}	200	10^{-3}	200	270	Si wafer
3	1.2: 0.7	63: 37	10^{-6}	200	10^{-3}	200	178	Si wafer
4	2.0: 0.7	74: 26	10^{-6}	200	10^{-3}	200	265	Si wafer
5	1.2: 0.5	71: 29	10^{-6}	200	10^{-3}	200	385	Si wafer
6	1.2: 0.5	71: 29	10^{-6}	400	10^{-3}	200	250	Si wafer
7	1.2: 0.5	71: 29	10^{-6}	400	10^{-3}	200	160	Si wafer, FS

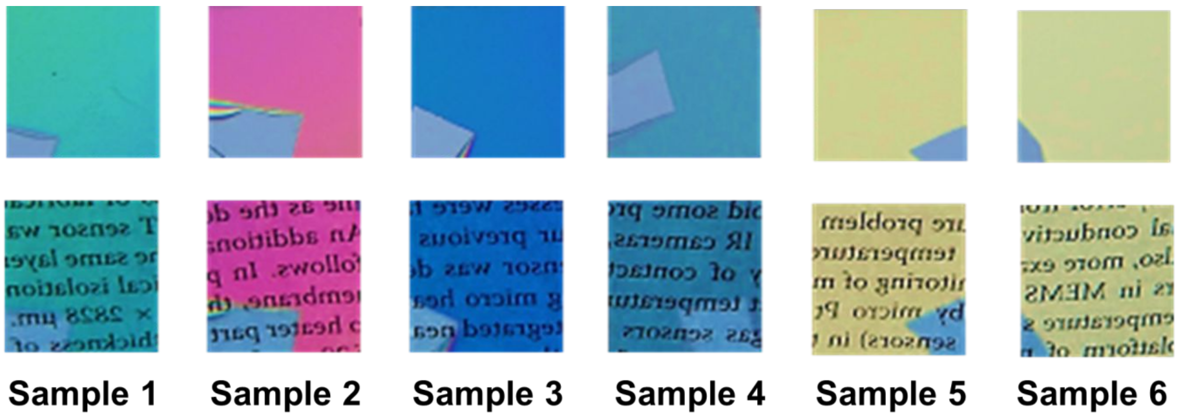

Figure S2. Photographs of mirror-like HfN thin films on 10 mm x 10 mm Si (100) substrates grown by reactive sputtering under varying Ar: N_2 flow rates.

Supporting Information

S2. XRD Analysis

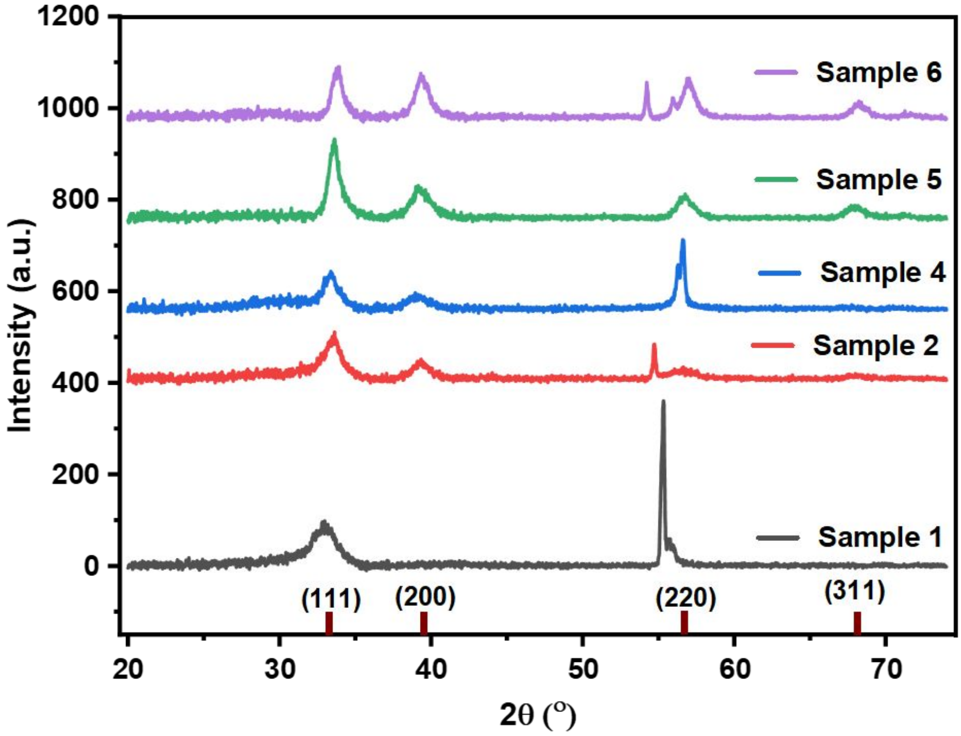

Figure S3. Grazing incidence x-ray diffraction (GIXRD) patterns of Samples 1 to 6 obtained at an incident angle of 3°. The diffraction peaks correspond to the (111), (200), (220), and (311).

S3. Raman Analysis

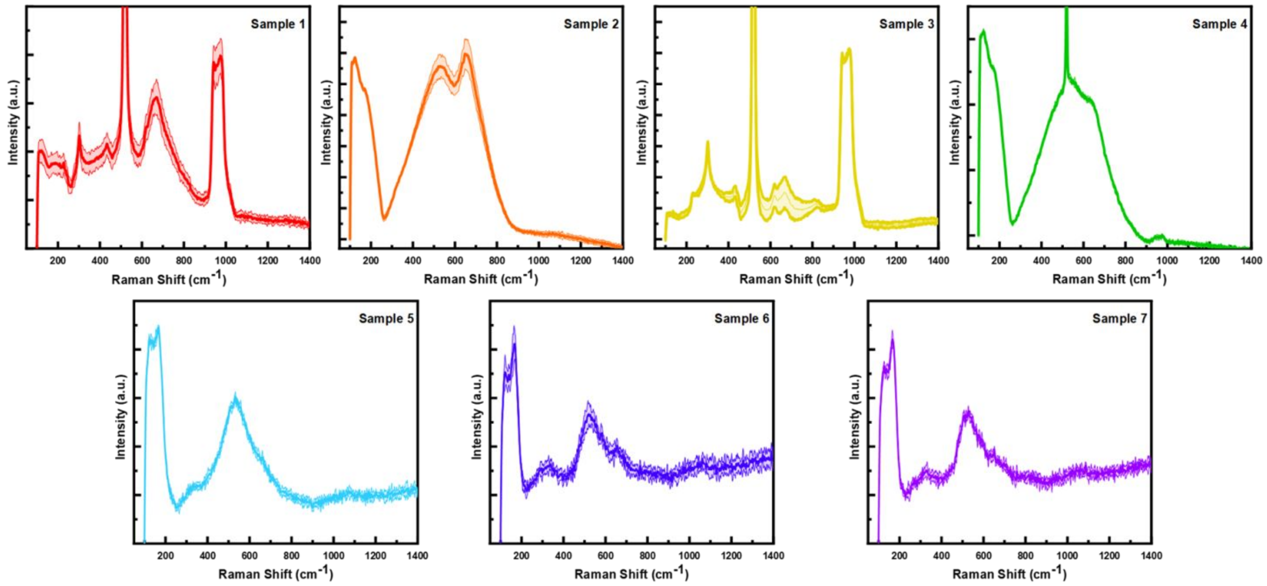

Figure S4. Raman spectra of Samples 1 to 7, showcasing the vibrational modes characteristic of the HfN's molecular structure. Each spectrum reveals distinct peaks corresponding to specific bond vibrations, indicating variations in the chemical composition. In the Raman spectra, the shaded region represents the standard deviation of measurements taken from five different points.

S4. Stoichiometric analysis of HfN thin films at varying Ar: N_2 ratios

Hafnium-based nitrides crystallize in a simple NaCl-type structure consisting of two interpenetrating sublattices: one comprised of metal (Hf) atoms and the other of non-metal (nitrogen) atoms [2]. Transition metal nitrides (TMNs) from group IVB typically exhibit a nominal 1:1 metal-to-nitrogen ratio; however, this phase is non-stoichiometric. The crystal structure exhibits a stoichiometric composition with a 3:4 metal-to-nitrogen ratio (e.g., Hf_3N_4), wherein each Hf atom contributes four valence electrons, while each nitrogen atom necessitates three electrons to fulfill its p orbital configuration. In these stoichiometric phases, complete electron transfer leads to semiconducting properties, as demonstrated by c-Hf_3N_4, which displays a notable band gap and is recognized for its transparency and electrical insulation. In contrast, metal-rich nitrides generally exhibit conductivity due to residual electrons in the metal d orbitals or the nitrogen p orbitals. The excess electrons contribute to the plasmonic and conductive properties of non-stoichiometric phases [3].

The main aim of this study was to deposit plasmonic HfN thin films. To attain plasmonic characteristics, we sputtered HfN thin films with diverse stoichiometries (Hf/N+O) by modulating the flow rates of Ar and N_2 gases during deposition [4], resulting in the observation of plasmonic thin films in non-stoichiometric HfN phases. Energy-dispersive X-ray spectroscopy (EDX) analysis indicated that the elemental composition of the thin films is directly related to the gas flow ratios employed during the deposition process. The ideal gas flow ratio for the deposition of plasmonic HfN thin films was established as 71% Ar to 29% N_2.

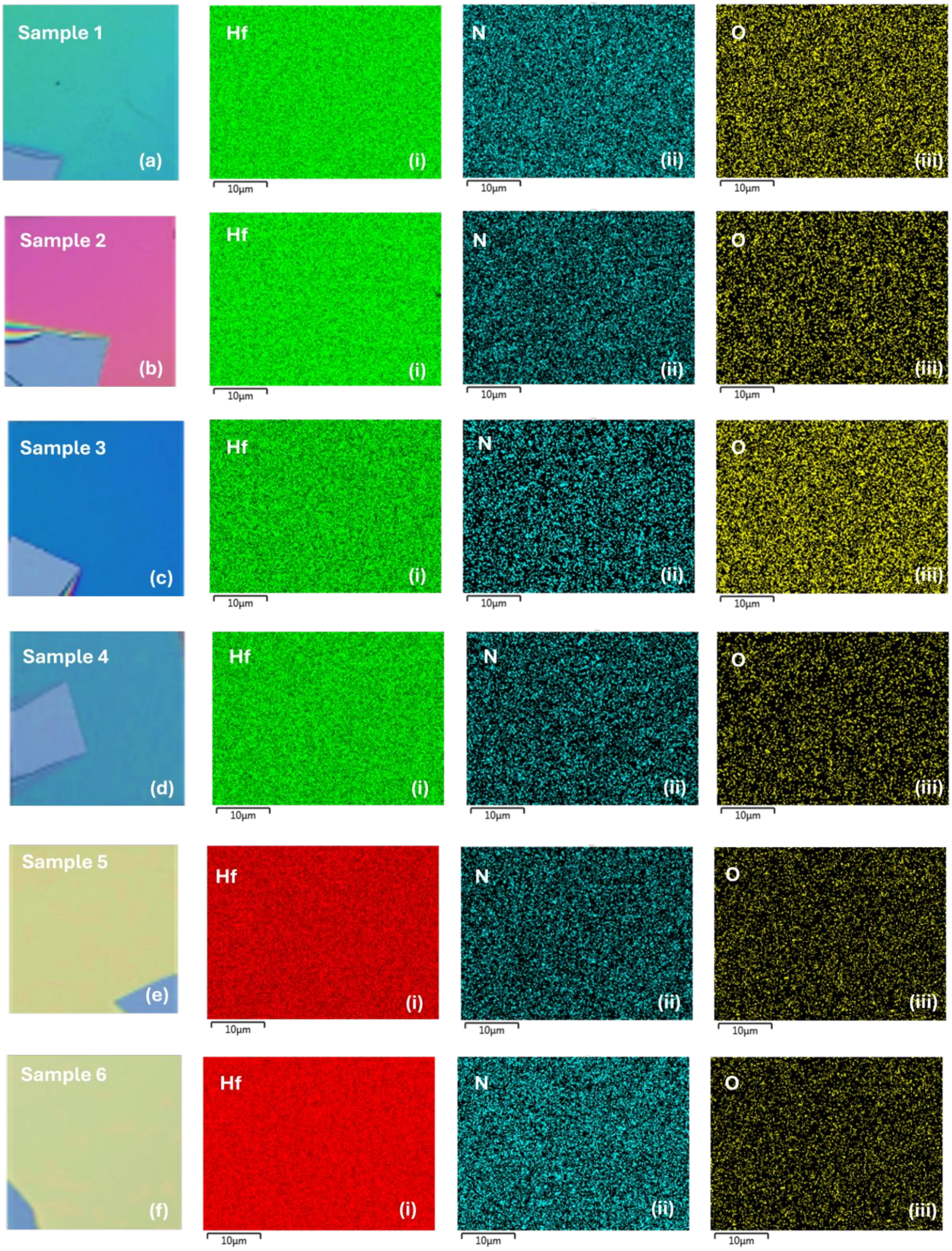

Figure S5. Photographs on HfN thin films on 10 mm x 10 mm Si (100) substrates at different Ar: N_2 ratios (sccm) obtained by SEM-EDX mapping analysis. The samples are labeled as Sample 1 (a), Sample 2 (b), Sample 3 (c), Sample 4 (d), Sample 5 (e), Sample 6 (f), respectively.

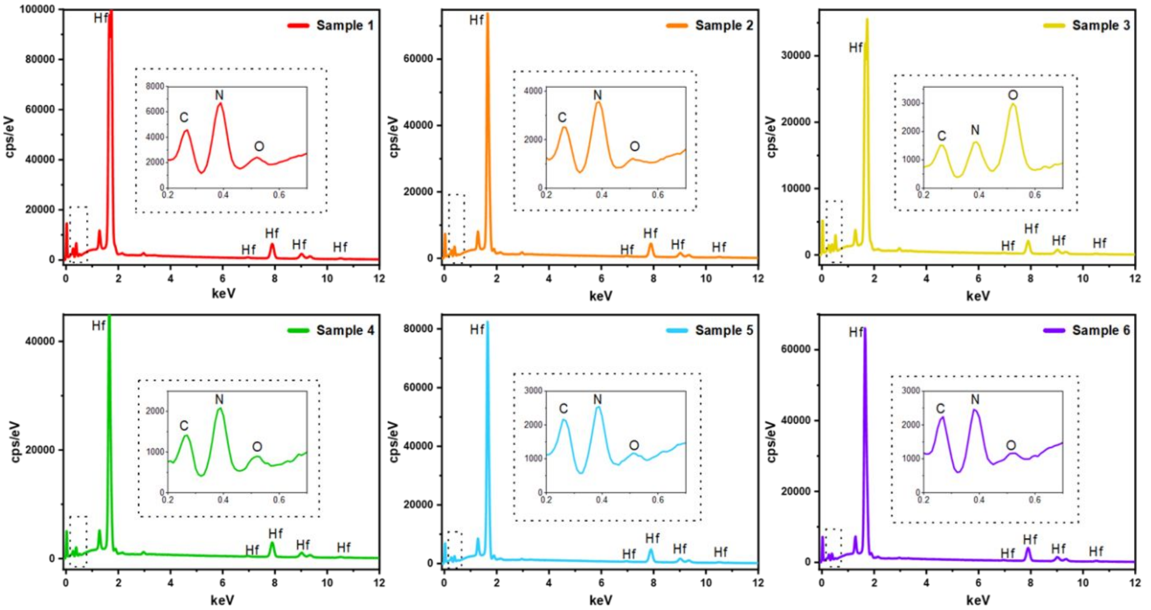

Figure S6. SEM-EDS analysis results for HfN thin films synthesized under varying deposition parameters. The elemental distributions are presented for each sample (Samples 1–6), with distinct peaks corresponding to Hf, N, traces of C and O detected within the thin film structures. Insets in each graph highlight the relative concentrations of these elements, emphasizing the elemental uniformity and potential impurities across the samples.

S5. Optical properties of HfN thin films at varying Ar:N_2 gas flow ratios

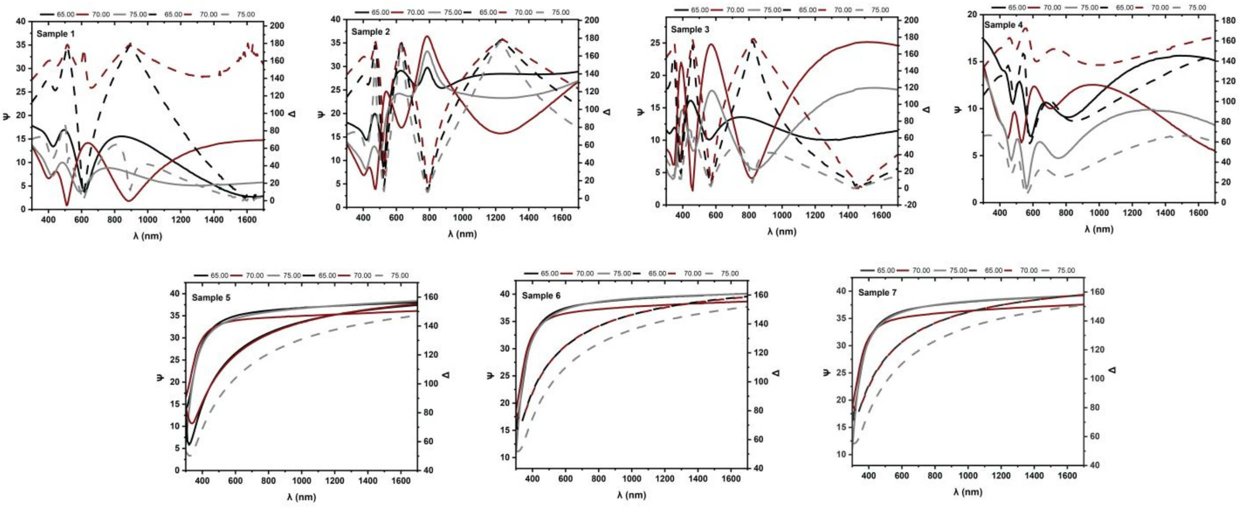

Figure S7. Ellipsometric Ψ (psi) and Δ (delta) spectra of HfN thin films deposited under varying sputter gas flow conditions, measured at incident angles of 65°, 70°, and 75°. The graphs illustrate the wavelength dependence of the optical parameters, revealing variations in film thickness, refractive index, and extinction coefficient. The distinct spectral features reflect differences in the structural and optical properties induced by the sputtering gas flow rates during deposition.

Supporting Information

S6. Ellipsometry Fitting

Table S2. Drude-Lorentz ellipsometry fitting parameters for HfN thin films produced under different sputter gas flow conditions. The table presents the mean square error (MSE), high-frequency dielectric constant (ε_∞), carrier concentration (N), unscreened plasma energy (E_{pu}), scattering time (τ), Drude broadening parameter (Γ_D), and Lorentz parameters, including oscillator strengths (f_1, f_2), resonant energies (E_1, E_2), and damping coefficients (γ_1, γ_2). These values reflect the electrical and optical properties of the films, showcasing variations in free carrier dynamics and interband transitions influenced by the sputtering gas flow rates.

Sample No	MSE	ε_∞	N (cm^{-3})	E_{pu} (eV)	τ (fs)	Γ_D (eV)	f_1	γ_1 (eV)	E_1 (eV)	f_2	γ_2 (eV)	E_2 (eV)
**5**	1.55	2.10	$2.07\cdot10^{22}$	5.3416	0.644	1.02	67.71	1.76	0.33	111.01	0.32	5.46
**6**	1.22	6.05	$2.25\cdot10^{22}$	5.5663	1.414	0.47	96.24	1.45	0.37	22.25	1.12	6.20
**7**	3.55	0.32	$5.84\cdot10^{21}$	2.8371	1.302	0.51	107.38	1.44	0.43	155.71	0.67	7.46

S7. Hall Measurement

Table S3. Hall measurement results for HfN thin films produced under different sputter gas flow conditions. The table summarizes bulk resistivity (Ω·cm), carrier concentrations (cm^{-3}), and carrier mobility (cm^2/V·s). Variations in these parameters highlight the influence of gas flow ratios on the electrical transport properties of the films, with significant differences in resistivity, free carrier density, and mobility.

Sample No	Ar: N_2 (%)	Bulk Resistivity [Ω. cm]	Carrier Concentration [cm^{-3}]	Mobility [cm^2 V^{-1} s^{-1})]
**2**	50:50	$1.91\cdot10^{-4} \pm 3.37\cdot10^{-8}$	$1.00\cdot10^{22} \pm 7.03\cdot10^{20}$	$3.89 \pm 5.28\cdot10^{-6}$
**4**	74:26	$7.39\cdot10^{-1} \pm 3.61\cdot10^{-3}$	$6.07\cdot10^{16} \pm 1.81\cdot10^{14}$	$0.14 \pm 2.21\cdot10^{-5}$
**5**	71:29	$7.37\cdot10^{-5} \pm 1.85\cdot10^{-9}$	$8.24\cdot10^{22} \pm 9.51\cdot10^{21}$	1.04 ± 0.104
**6**	71:29	$3.60\cdot10^{-5} \pm 2.73\cdot10^{-7}$	$1.87\cdot10^{22} \pm 4.82\cdot10^{21}$	10.0 ± 2.79
**7**	71:29	$7.78\cdot10^{-5} \pm 1.30\cdot10^{-8}$	$1.83\cdot10^{23} \pm 1.49\cdot10^{22}$	0.44 ± 0.04

Supporting Information

S8. Literature Comparison

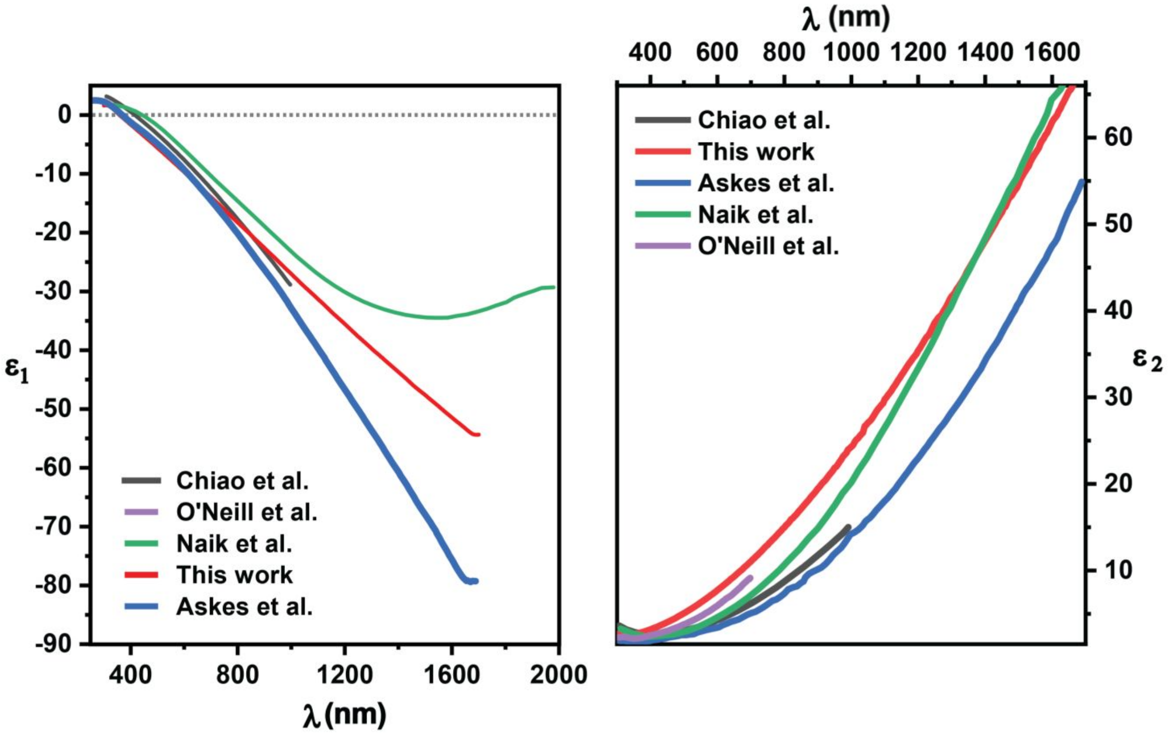

Figure S8. Comparison of the real (ε_1) and imaginary (ε_2) parts of the dielectric function as a function of λ for the current work and previously published studies (Chiao et al.,[5] O'Neill et al.,[6] Naik et al.,[7] and Askes et al..[8] The left panel shows the real part, transitioning from positive to negative values, indicating the material's plasmonic behavior. The right panel illustrates the imaginary part, highlighting optical losses. The results demonstrate the alignment and deviations of the current findings with existing literature.

Supporting Information

S9. XPS Analysis

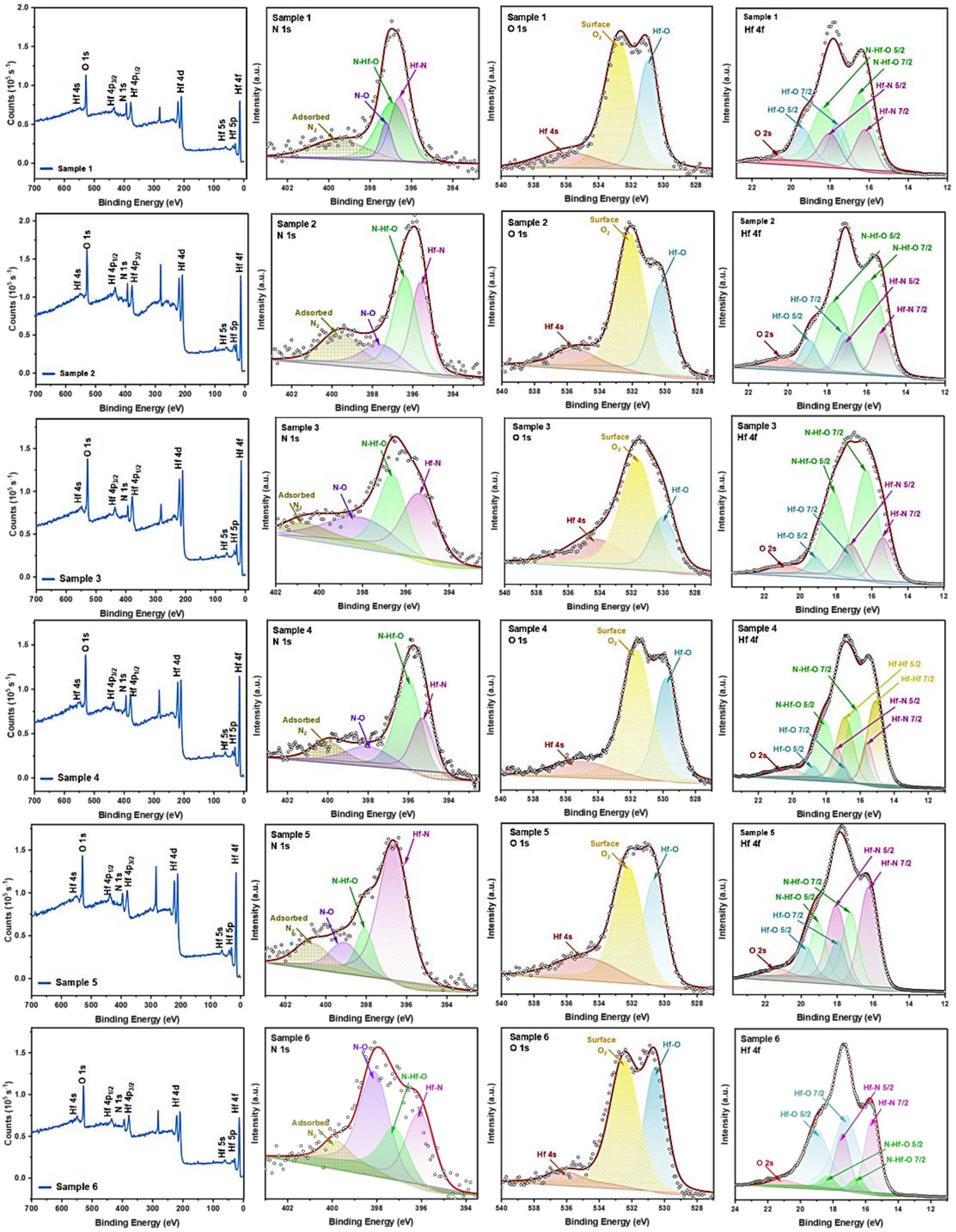

Figure S9. X-ray photoelectron spectroscopy (XPS) results of HfN thin films produced under varying sputter gas flow conditions. The spectra include core-level analysis for N 1s, O 1s, and Hf 4f regions, with deconvoluted peaks identifying contributions from Hf–N, Hf–O, and surface-adsorbed species such as NO_x and O_2. The variations in peak positions, intensities, and deconvoluted components highlight the influence of gas flow on chemical bonding, surface oxidation, and nitrogen incorporation in the films. These results provide insights into the compositional and chemical states of the films under different deposition conditions.

S10. Sample 7 (EBL Sample) data explanation for HfN thin film on Si wafer and FS substrate

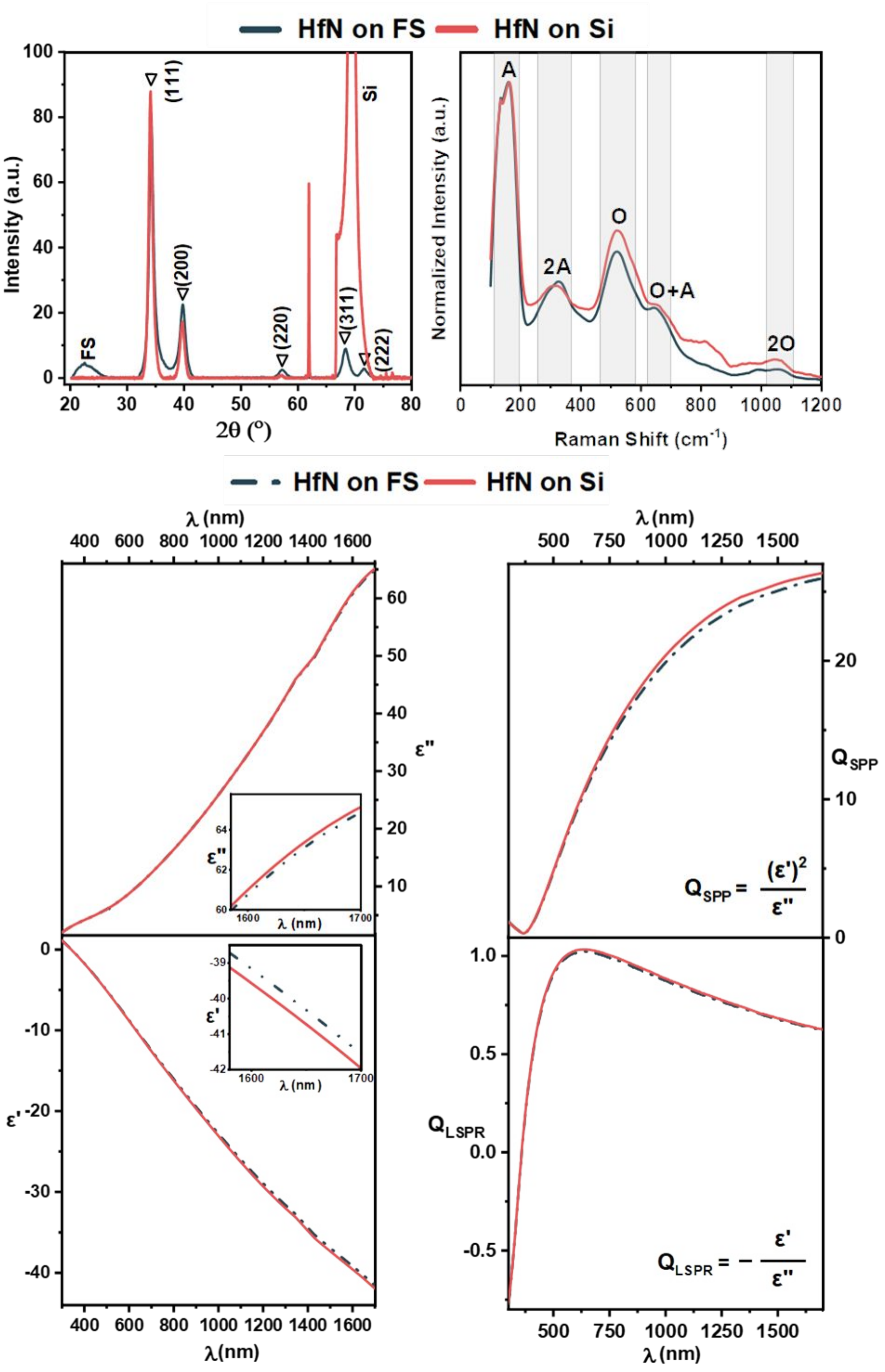

Figure S10. Analysis of HfN thin films (Sample 7) deposited on Si wafer and fused silica (FS) substrates used EBL for HfN nanocavity and nanodisk arrays. The XRD patterns (top left) highlight the crystalline orientations of HfN on both substrates, with distinct peak intensities reflecting substrate influence. The Raman spectra (top right) reveal vibrational modes, including A, O, and O+A peaks, showcasing differences in structural and bonding characteristics. The bottom plots illustrate the wavelength-dependent real (ε') and imaginary (ε'') parts of the dielectric function, along with quality factors for surface plasmon polaritons (Q_{SPP}) and localized surface plasmon resonances (Q_{LSPR}), emphasizing variations in optical and plasmonic properties between the two substrates.

Supporting Information

S11. FDTD simulations for HfN nanocavity array

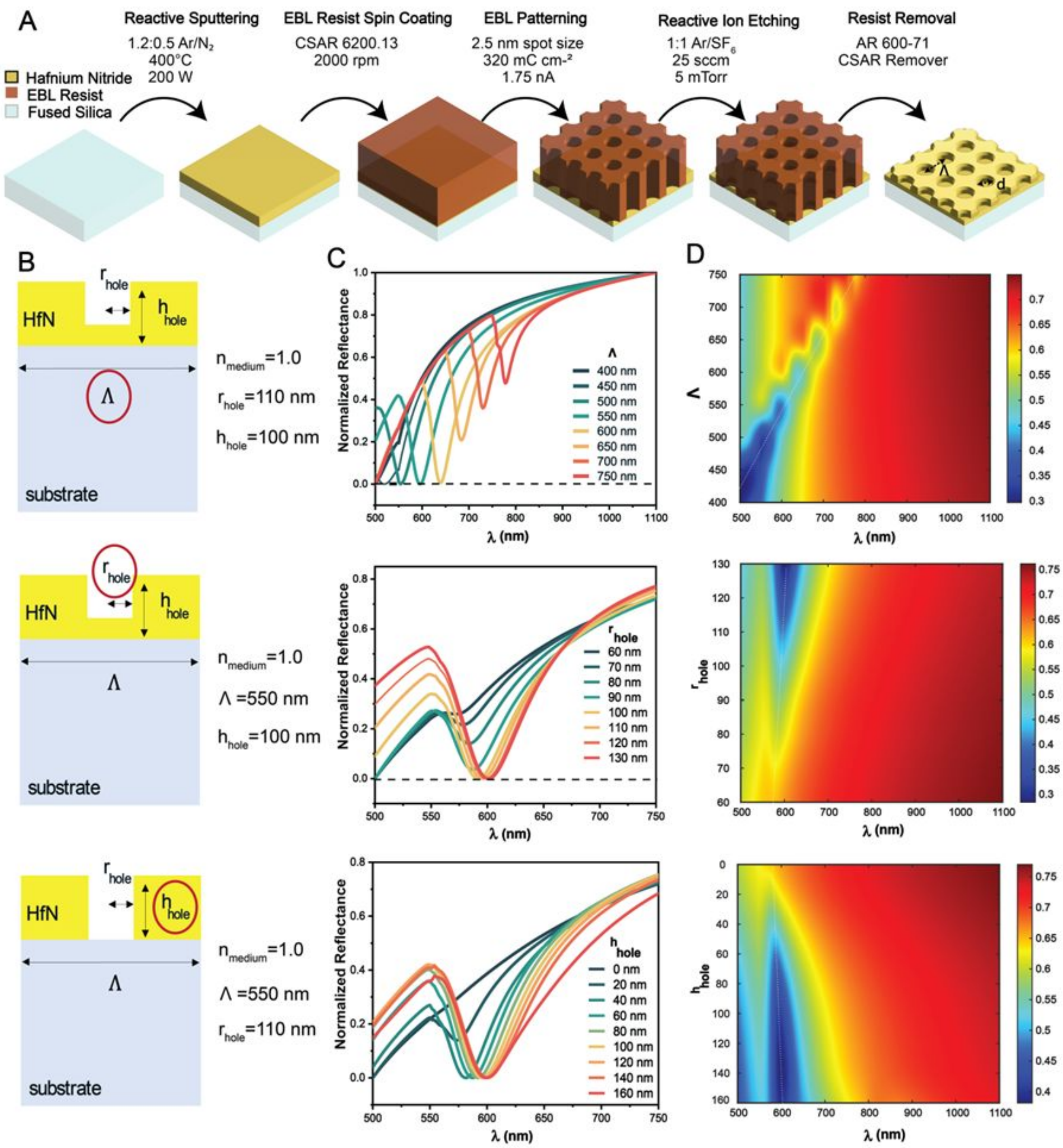

Figure S11. The top of the figure represents the schematic fabrication process for HfN nanocavity array, including reactive sputtering, EBL resist spin coating, EBL patterning, reactive ion etching, and resist removal (A). The structural simulation parameters: Lattice periods (Λ), hole radius (r_{hole}), and hole depths (h_{depth}) (B); the normalized reflectance spectra for various Λ, r_{hole}, and h_{depth} of HfN nanocavity array are shown under air ambient conditions (C); the right panel displays detailed reflectance heatmaps (D). These heatmaps plot the reflectance intensity as a function of wavelength and varying lattice period, hole radius, or hole depth, providing a comprehensive visualization of how the optical response varies with structural parameters.

S12. The surface topology of bare HfN film and patterned plasmonic HfN nanocavity array by AFM analysis.

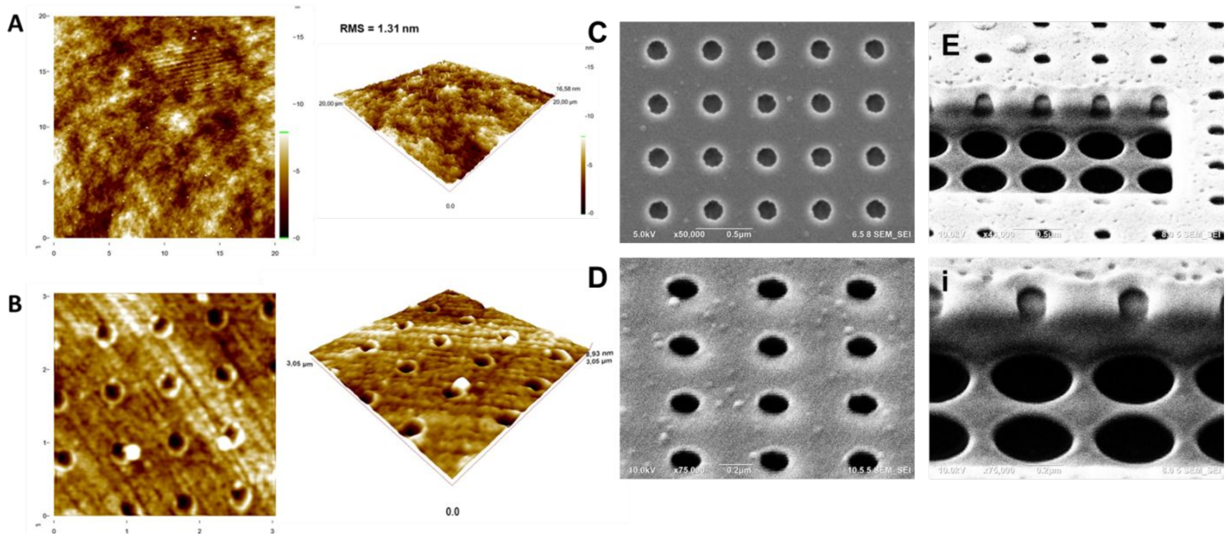

Figure S12. Surface topography by tapping mode AFM analysis. A) The surface topography of the bare Sample 6 had a low level of roughness (1.31 nm root-mean-square(RMS) B) The AFM images of HfN nanocavity arrays represented with a scale of 3.0 µm × 3.0 µm. Morphologic images C), D), and E) are two-dimensional, angled, and sidewall images acquired using focused ion beam scanning electron microscopy (FIB-SEM), respectively.

S13. Refractometric sensing results of HfN nanostructures at various glycerol concentrations

Table S4. Refractive index calculations for glycerol-water solutions at varying glycerol concentrations (wt.%) based on the Brix values at 20°C. The data demonstrate the glycerol concentration, Brix value, and refractive index, providing essential information for the optical characterization of glycerol-water concentrations.

**Glycerol Concentrations (wt.%)**	**Brix Value at 20°C**	**n_{medium} $n=1.332+(0.0015\times Brix)+(2.8\times10^{-6}\times Brix^2)$**
**1**	1	1.333503
**3**	2	1.335011
**5**	5	1.33957
**10**	10	1.34728
**20**	18.5	1.360708
**30**	25	1.37125
**40**	33	1.384549
**50**	39.5	1.395619

Supporting Information

Table S5. The plasmonic resonance wavelength values of HfN nanocavity arrays at various glycerol (GLY) concentrations for a period of 500 nm.

**Array**	**Water**	**1.0 wt.% GLY**	**3.0 wt.% GLY**	**5.0 wt.% GLY**	**10.0 wt.% GLY**	**20.0 wt.% GLY**	**30.0 wt.% GLY**	**40.0 wt.% GLY**	**50.0 wt.% GLY**
1	736.94	742.3	738.55	742.04	744.13	748.77	754.93	761.83	767.32
2	742.37	743.93	743.18	745.87	746.64	750.85	757.21	764.05	770.66
3	732.57	738.11	739.24	737.7	741.06	749.72	752.23	757.35	766.4
4	727.01	729.33	730.08	732.58	733.27	739.23	744.54	751.6	759.23
5	735.83	737.73	737.2	740.16	739.82	744.42	751.68	758.39	764.74
6	733.41	735.3	734.37	737.43	739.95	742.81	749.01	757	764.3
7	729.96	731.53	732.59	735.71	735.84	738.77	746.46	753.05	762.56
8	729.12	733.56	732.64	733	736.29	736.71	747.07	754.01	761.5
Mean	731.3167	734.26	734.3533	736.0967	737.705	741.9433	748.4983	755.2333	763.1217
STD.	3.210493	3.469663	3.35528	3.35528	3.030477	4.736698	3.038864	2.721416	2.563017

Table S6. The plasmonic resonance wavelength values of HfN nanocavity arrays at various glycerol concentrations for a period of 550 nm.

**Array**	**Water**	**1.0 wt.% GLY**	**3.0 wt.% GLY**	**5.0 wt.% GLY**	**10.0 wt.% GLY**	**20.0 wt.% GLY**	**30.0 wt.% GLY**	**40.0 wt.% GLY**	**50.0 wt.% GLY**
1	785.1	788.57	787.45	789.63	796.33	799.57	806.13	815.35	825.61
2	783.53	785.64	787.86	789.88	792.39	798.3	804.58	812.64	817.65
3	787.45	788.23	785.88	788.62	790.88	799.78	802.13	808.79	824.18
4	782.42	782.25	788.69	786.53	785.87	793.15	800.55	806.88	820.17
5	784.73	782.66	784.2	785.7	789.84	792.88	802.1	809.24	816.65
6	781.77	782.42	786.47	786.08	787.48	798.76	800.51	808.44	822.55
7	780.35	784.82		788.21	786.34	796.7	802.6	808.9	815.36
8	780.41	783.14		786.62	787.91	792.93	800.44	807.14	830.18
Mean	781.936	783.058	786.4533	786.628	787.488	794.884	801.24	808.12	820.982
STD.	1.796686	1.040394	2.245046	0.958368	1.552891	2.700098	1.029344	1.056314	5.875676

Supporting Information

Table S7. The plasmonic resonance wavelength values of HfN nanocavity arrays at various glycerol concentrations for a period of 600 nm.

**Array**	**Water**	**1.0 wt.% GLY**	**3.0 wt.% GLY**	**5.0 wt.% GLY**	**10.0 wt.% GLY**	**20.0 wt.% GLY**	**30.0 wt.% GLY**	**40.0 wt.% GLY**	**50.0 wt.% GLY**
1	841.41	845.69	846.64	848.82	850.6	858.43	867.2	874.92	885.85
2	842.75	845.85	847.89	848.04	850.46	858.16	866.51	873.89	886.94
3	846.53	847.48	846.21	851.57	852.92	857.64	866.93	877.15	889.08
4	845.25	846.05	846.3	848.65	850.43	863.65	866.99	877.05	890.89
5	842.5	846.84	847.34	850.58	850.83	857.46	867.36	876.88	886.87
6	847.57	845.98	848.37	851.41	851.42	858.22	867.47	878.71	885.76
7	844.3	848.98	848.84	852.04	851.85		868.72	879.64	885.58
Mean	844.905	846.962	847.412	850.67	851.1325	859.7767	867.635	878.07	887.636
STD.	2.111185	1.40039	1.1878	1.1878	0.62782	3.37586	0.751909	1.333042	2.291665

Table S8. The refractive index sensitivity of plasmonic HfN nanocavity arrays with periods of 500, 550, and 600 nm for the varying refractive indices. The spectral measurements were obtained from eight identically constructed 200 µm × 200 µm nanocavity arrays. The sensitivity was calculated from the slope of the linear regression according to the center wavelength vs. refractive index.

	Arrays	a	B1 (Slope)	R Square
**P500**	1	1.46161E2	4.44301E2	0.97733
	2	1.77419E2	4.23536E2	0.97721
	3	1.23153E2	4.59433E2	0.96603
	4	1.06855E2	4.65921E2	0.98494
	5	1.60161E2	4.31775E2	0.9724
	6	1.27662E2	4.54403E2	0.97705
	7	1.19943E2	4.57874E2	0.95774
	8	1.21203E2	4.56705E2	0.93736
**P550**	1	8.31968	5.83418E2	0.9803
	2	9.91025E1	5.14715E2	0.99263
	3	8.46156E1	5.25684E2	0.94517
	4	8.50999E1	5.22915E2	0.93075
	5	9.74992E1	5.13892E2	0.97935
	6	1.93694E1	5.7205E2	0.96087
	7	8.84278E1	5.20462E2	0.97831
	8	-8.91396E1	6.51687E2	0.9011
**P600**	1	-4.0764	6.35654E2	0.98364
	2	9.52354	6.25699E2	0.97438
	3	4.828	6.30393E2	0.96756
	4	-4.0764	6.30393E2	0.98364
	5	-3.73154	6.35981E2	0.97439
	6	3.40799E1	6.08768E2	0.97499
	7	1.90296E1	6.20581E2	0.98605
	8	1.01925E2	5.57305E2	0.9088

Supporting Information

Table S9. The plasmonic resonance wavelength values of HfN nanodisk arrays at various glycerol concentrations for a period of 500 nm.

**Array**	**Water**	**1.0 wt.% GLY**	**3.0 wt.% GLY**	**5.0 wt.% GLY**	**10.0 wt.% GLY**	**20.0 wt.% GLY**	**30.0 wt.% GLY**	**40.0 wt.% GLY**	**50.0 wt.% GLY**
1	777.76	780.87	786.44	788.55	793.65	797.37	804.15	808.32	811.47
2	778.64	782.44	787.43	791.09	796.94	801.71	807.65	807.43	812.43
3	778.34	781.31	786.44	790.3	792.75	798.3	804.15	808.32	812.43
4	777.46	780.86	784.62	792.83	795.29	800	805.83	810.84	812.43
5	777.76	781.09	789.24	789.35	793.65	801.71	806.6	809.95	812.43
6	778.64	785.81	787.43	791.09	794.39	800.93	804.15	809.05	811.48
7	778.29	783.34	784.62	789.35	792.75	799.08	805.83	809.95	812.1
8	779.09	785	787.43	791.09	794.4	800	804.91	809.96	813.38
Mean	778.2475	782.5794	786.7063	790.4563	794.2275	799.8875	805.4088	809.2275	812.2688
STD.	0.467714809	1.824069	1.645162	1.442589	1.502317	1.695616	1.38534	1.188637	0.450222

Table S10. The plasmonic resonance wavelength values of HfN nanodisk arrays at various glycerol concentrations for a period of 550 nm.

**Array**	**Water**	**1.0 wt.% GLY**	**3.0 wt.% GLY**	**5.0 wt.% GLY**	**10.0 wt.% GLY**	**20.0 wt.% GLY**	**30.0 wt.% GLY**	**40.0 wt.% GLY**	**50.0 wt.% GLY**
1	817.17	818.8	822.5	824.82	830.73	831.64	836.58	840.81	854.92
2	816.31	820.59	823.56	825.37	830.48	831.38	834.62	841.74	854.38
3	816.54	819.94	822.93	824.03	831.43	833.06	835.48	840.3	855.92
4	816.31	820.39	824.6	824.06	831.63	833.32	835.72	841.23	853.93
5	815.89	819.67	823.61	827.87	831.43	834.26	836.38	842.68	854.38
6	816.31	818.8	823.5	825.03	831.94	834.26	836.58	841.74	855.47
7	817.16	819.25	822.57	826.07	831.24	835.2	837.26	840.81	854.38
8	816.74	818.8	822.36	827.07	833.22	834.73	837.69	841.75	853.39
Mean	816.55375	819.53	823.2038	825.54	831.5125	833.4813	836.2888	841.3825	854.5963
STD.	0.476330218	0.720991	0.730225	1.332497	0.507656	1.41145	0.875889	0.792717	0.70674

Table S11. The plasmonic resonance wavelength values of HfN nanodisk arrays at various glycerol concentrations for a period of 600 nm.

**Array**	**Water**	**1.0 wt.% GLY**	**3.0 wt.% GLY**	**5.0 wt.% GLY**	**10.0 wt.% GLY**	**20.0 wt.% GLY**	**30.0 wt.% GLY**	**40.0 wt.% GLY**	**50.0 wt.% GLY**
1	876.89	880.06	882.31	886.95	888.73	891.47	892.61	892.82	905.41
2	875.71	881.79	882.82	886.78	888.2	890.43	893.03	893.38	905.96
3	877.8	881.11	884.15	886.31	889.15	890.05	891.85	894.4	905.05
4	878.72	880.43	884.65	886	888.72	890.76	893.03	894.96	903.95
5	877.81	880.06	883.65	886.62	889.67	890.05	892.61	895.99	905.05

6	877.48	880.74	884.15	885.66	890.09	891.15	891.85	894.96	905.96
7	879.31	881.42	883.74	886.12	888.73	888.24	891.43	894.59	905.05
8	878.4	880.74	883.23	887.95	890.01	888.94	890.67	895.99	905.96
Mean	877.765	880.7938	883.5875	886.5481	888.38	890.1363	892.135	894.6363	905.2988
STD.	1.177183887	0.670359	0.815648	0.459455	0.647004	1.055789	0.633268	1.057934	0.686679

S14. Au nanodisk array refractometric performance

Table S12. The plasmonic resonance wavelength values of Au nanodisk arrays at various glycerol concentrations for a period of 500 nm.

**Array**	**1.0 wt.% GLY**	**3.0 wt.% GLY**	**5.0 wt.% GLY**	**10.0 wt.% GLY**	**20.0 wt.% GLY**	**30.0 wt.% GLY**	**40.0 wt.% GLY**	**50.0 wt.% GLY**
1	753.88	755.67	755.16	755.59	759.24	761.79	764.75	772.84
2	753.18	755.98	755.31	755.44	759.24	761.92	763.71	772.6
3	751.27	754.37	754.26	755.11	760.03	760.44	762.7	771.32
4	750.71	753.26	753.84	753.77	758.09	759.72	761.79	770.52
5	753.48	755.55	756.87	753.77	758.21	761.41	763.56	773.88
6	754.88	756.48	757.06	755.33	759.19	762.17	764.5	773.88
7	753.48	756.58	756.07	755.33	760.99	762.17	763.71	772.97
8	754	755.34	756.79	755.59	760.34	762.37	764.96	772
Mean	753.1	755.4038	755.67	754.9913	759.4163	761.4988	763.71	772.5013
STD.	1.47405	1.199301	1.199301	0.789005	1.004886	0.944084	1.017471	1.253724

Table S13. The plasmonic resonance wavelength values of Au nanodisk arrays at various glycerol concentrations for a period of 550 nm.

**Array**	**1.0 wt.% GLY**	**3.0 wt.% GLY**	**5.0 wt.% GLY**	**10.0 wt.% GLY**	**20.0 wt.% GLY**	**30.0 wt.% GLY**	**40.0 wt.% GLY**	**50.0 wt.% GLY**
1	805.12	803.85	804.88	804.96	806.19	810.42	812.09	822.47
2	803.66	805.38	803.95	804.88	805.15	809.27	811.57	821.6
3	802.76	803.38	803.65	804.08	804.62	809.74	811.85	820.58
4	804.22	804.37	804.24	805.73	805.15	810.89	811.33	820.81
5	803.8	803.57	803.6	806.13	807.41	810.88	812.57	820.95
6	804.85	805.9	805.57	806.35	806.1	812.06	813.33	820.54
7	803.94	806.61	807.49	805.91	810.46	811.88	814.52	822.83
8		805.03	805.63		808.02	810.06	813.19	822.24
Mean	804.05	804.7613	804.8763	805.4343	806.6375	810.65	812.5563	821.5025
STD.	0.785769	0.785769	1.392066	0.816759	1.931112	1.12475	1.12475	1.12475

Table S14. The plasmonic resonance wavelength values of Au nanodisk arrays at various glycerol concentrations for a period of 600 nm.

Supporting Information

Array	1.0 wt.% GLY	3.0 wt.% GLY	5.0 wt.% GLY	10.0 wt.% GLY	20.0 wt.% GLY	30.0 wt.% GLY	40.0 wt.% GLY	50.0 wt.% GLY
1	864.09	864.6	866.39	866.01	867.07	872.68	872.83	884.62
2	863.8	865.8	866.6	867.42	867.11	871.27	872.37	884.72
3	864.34	865.28	866.76	866.36	866.79	873.42	871.74	885.57
4	864.19	865.42	866.53	867.4	867.55	871.78	871.43	885.88
5	864.34	866.17	866.6	867.11	867.29	872.33	872.84	886.33
6	863.8	865.48	865.59	866.42	867.56	870.88	872.6	886.12
7	864.93	865.42	866.38	865.77	867.65	872.14	872.83	886.23
8		866.3	867.28	867.23	867.56	871.27	872.83	
Mean	864.2129	865.5588	866.5163	866.715	867.3225	871.9713	872.4338	885.6386
STD.	0.387974	0.482173	0.383567	0.653627	0.857058	0.857058	0.857058	0.707565

S15. The Coupled Dipole Approximation (CDA)

$$P_i = a_i a E_{loc,i} \quad \text{(S1)}$$

$$E_{loc,i} = E_{inc,i} + E_{dipole,i} = E_0 \exp(ik.r_i) - \sum_{j=1}^{N} A_{ij}.P_j (i = 1,2,\ldots,N) \,, j \neq 1 \quad \text{(S2)}$$

E_0 is amplitude and $k = {}^{2\pi}/_{\lambda}$ is wavenumber of incident wave.

$$A_{ij}.P_j = k^2 \exp\left(ik.r_{ij}\right) \frac{r_{ij} \times \left(r_{ij} \times P_j\right)}{r_{ij}^3} + \exp\left(ik.r_{ij}\right)\left(1 - ik.r_{ij}\right) \frac{\left[r_{ij}^2 P_j - 3r_{ij}\left(r_{ij}.P_j\right)\right]}{r_{ij}^5} \quad \text{(S3)}$$

$$P = \frac{E_0}{\frac{1}{\alpha_s} - S} \quad \text{(S4)}$$

$$C_{ext} = 4\pi k \, Im\left(\frac{P}{E_0}\right) \quad \text{(S5)}$$

Supporting Information

$$S = \sum_{j\#1} \left(\frac{(1-ik.r_{ij})(3cos^2\theta_{ij}-1)\exp(ik.r_{ij})}{r_{ij}^3} + \frac{k^2 sin^2\theta_{ij}\exp(ik.r_{ij})}{r_{ij}} \right) \quad (S6)$$

References

(1) Günaydın, B. N.; Gülmez, M.; Torabfam, M.; Pehlivan, Z. S.; Tütüncüoğlu, A.; Kayalan, C. I.; Saatçioğlu, E.; Bayazıt, M. K.; Yüce, M.; Kurt, H. Plasmonic Titanium Nitride Nanohole Arrays for Refractometric Sensing. *ACS Appl Nano Mater* **2023**, *6* (22), 20612–20622. https://doi.org/10.1021/acsanm.3c03050.

(2) Razumovskiy, V. I.; Popov, M. N.; Ding, H.; Odqvist, J. Formation and Interaction of Point Defects in Group IVb Transition Metal Carbides and Nitrides. *Comput Mater Sci* **2015**, *104*. https://doi.org/10.1016/j.commatsci.2015.03.042.

(3) Xie, X.; Wen, M.; Dong, H.; Long, H.; Zhang, X.; Wu, F.; Mu, Z. Semiconductors with a Chiral Crystal Structure in Group IVB Transition Metal Pernitrides. *Physical Chemistry Chemical Physics* **2022**, *24* (36). https://doi.org/10.1039/d2cp02627a.

(4) Judek, J.; Dhama, R.; Pianelli, A.; Wróbel, P.; Michałowski, P. P.; Dana, J.; Caglayan, H. Ultrafast Optical Properties of Stoichiometric and Non-Stoichiometric Refractory Metal Nitrides TiNx, ZrNx, and HfNx. *Opt Express* **2024**, *32* (3). https://doi.org/10.1364/oe.505442.

(5) Chiao, Z.-Y.; Chen, Y.-C.; Chen, J.-W.; Chu, Y.-C.; Yang, J.-W.; Peng, T.-Y.; Syong, W.-R.; Lee, H. W. H.; Chu, S.-W.; Lu, Y.-J. Full-Color Generation Enabled by Refractory Plasmonic Crystals. *Nanophotonics* **2022**, *11* (12), 2891–2899. https://doi.org/10.1515/nanoph-2022-0071.

(6) O'Neill, D. B.; Frehan, S. K.; Zhu, K.; Zoethout, E.; Mul, G.; Garnett, E. C.; Huijser, A.; Askes, S. H. C. Ultrafast Photoinduced Heat Generation by Plasmonic HfN Nanoparticles. *Adv Opt Mater* **2021**, *9* (19). https://doi.org/10.1002/adom.202100510.

(7) Naik, G. V.; Kim, J.; Boltasseva, A. Oxides and Nitrides as Alternative Plasmonic Materials in the Optical Range [Invited]. *Opt Mater Express* **2011**, *1* (6). https://doi.org/10.1364/ome.1.001090.

(8) Askes, S. H. C.; Schilder, N. J.; Zoethout, E.; Polman, A.; Garnett, E. C. Tunable Plasmonic HfN Nanoparticles and Arrays. *Nanoscale* **2019**, *11* (42), 20252–20260. https://doi.org/10.1039/C9NR07683B.
